# DNAJA1- and conformational mutant p53-dependent inhibition of cancer cell migration by a novel compound identified through a virtual screen

**DOI:** 10.1038/s41420-022-01229-5

**Published:** 2022-10-31

**Authors:** Shigeto Nishikawa, Atsushi Kaida, Alejandro Parrales, Atul Ranjan, Mohamed Alalem, Hongyi Ren, Frank J. Schoenen, David K. Johnson, Tomoo Iwakuma

**Affiliations:** 1 Department of Pediatrics, Division of Hematology & Oncology, Children’s Mercy Research Institute, Kansas City, MO USA; 2 Department of Dental Radiology and Radiation Oncology, Graduate School of Medical and Dental Sciences, Tokyo Medical and Dental University, Tokyo, Japan; 3 Department of Cancer Biology, University of Kansas Medical Center, Kansas City, KS USA; 4 Higuchi Biosciences Center, University of Kansas, Lawrence, KS USA; 5 Molecular Graphics and Modeling Laboratory, University of Kansas, Lawrence, KS USA

**Keywords:** Tumour-suppressor proteins, Drug development

## Abstract

Cancers are frequently addicted to oncogenic missense mutant p53 (mutp53). DNAJA1, a member of heat shock protein 40 (HSP40), also known as J-domain proteins (JDPs), plays a crucial role in the stabilization and oncogenic activity of misfolded or conformational mutp53 by binding to and preventing mutp53 from proteasomal degradation. However, strategies to deplete mutp53 are not well-established, and no HSP40/JDPs inhibitors are clinically available. To identify compounds that bind to DNAJA1 and induce mutp53 degradation, we performed an in silico docking study of ~10 million of compounds from the ZINC database for the J-domain of DNAJA1. A compound 7-3 was identified, and its analogue A11 effectively reduced the levels of DNAJA1 and conformational mutp53 with minimal effects on the levels of wild-type p53 and DNA-contact mutp53. A11 suppressed migration and filopodia formation in a manner dependent on DNAJA1 and conformational mutp53. A mutant DNAJA1 with alanine mutations at predicted amino acids (tyrosine 7, lysine 44, and glutamine 47) failed to bind to A11. Cells expressing the mutant DNAJA1 became insensitive to A11-mediated depletion of DNAJA1 and mutp53 as well as A11-mediated inhibition of cell migration. Thus, A11 is the first HSP40/JDP inhibitor that has not been previously characterized for depleting DNAJA1 and subsequently conformational mutp53, leading to inhibition of cancer cell migration. A11 can be exploited for a novel treatment against cancers expressing conformational mutp53.

## Introduction

Targeting cancer-specific events is crucial for efficient anti-cancer therapies with minimal side effects [1]. Mutations in the tumor suppressor p53 are one of the most frequent cancer-specific events [2, 3]. Most p53 mutations are missense mutations, resulting in loss of function (LOF) as a tumor suppressor, as well as gain of new oncogenic activities (gain of function: GOF), which cannot simply be explained by p53 LOF [4, 5]. Accumulation of mutant p53 (mutp53) is central to displaying the oncogenic GOF [6–9]. High levels of mutp53 are frequently detected in human cancers, which is correlated with poor outcomes [10–13], while depletion of mutp53 inhibits malignant progression of cancer cells [14–17], suggesting the key role of mutp53 in cancer progression and addiction of cancers to mutp53. However, direct targeting and depletion of missense mutp53 have been challenging [4, 5].

We recently published that misfolded or conformational mutp53 interacts with and is stabilized by DNAJA1, a member of heat shock protein 40 (HSP40), also known as J-domain proteins (JDPs) [17–19]. HSP40/JDPs are involved in protein translation, folding/unfolding/refolding, and stabilization/degradation [20–22]. Clinically, DNAJA1 protein levels are increased in human head and neck cancers, which are correlated with reduced overall survival [18]. Knockdown of DNAJA1 triggers CHIP/STUB1 ubiquitin ligase-mediated proteasomal degradation of mutp53, leading to reduced malignant properties of cancer cells; however, knockdown of DNAJA1 has little effect on the levels of wild-type p53 (wtp53) and DNA-contact mutp53 [17, 18]. Thus, DNAJA1 is a conformational mutp53-dependent tumor-promoting factor, and mutp53 can be depleted by inhibiting DNAJA1. However, no DNAJA1- or HSP40/JDPs-specific inhibitors are clinically available.

We hypothesize that compounds which bind to and inhibit DNAJA1 would suppress malignant properties of cancer cells via depletion of conformational mutp53. To test this hypothesis, we performed an in silico docking study for the J-domain of DNAJA1 using the ZINC database with ~10 million of commercially available compounds, which identified a compound “7-3” (3-(4-hydroxy-3-methoxyphenyl)-3-(3-hydroxy-6-methyl-4-oxo-4H-pyran-2-yl)-N-(3-phenylpropyl)propanamide). Its analogue “A11” (3-(4-hydroxy-3-methoxyphenyl)-3-(3-hydroxy-6-methyl-4-oxo-4H-pyran-2-yl)-N-{3-[methyl(phenyl)amino]propyl}propanamide) efficiently depleted DNAJA1 and subsequently conformational mutp53 with minimal effects on the levels of DNA-contact mutp53 and wtp53. We demonstrate DNAJA1- and conformational mutp53-dependent activities of A11.

## Results

### A virtual screen identifies a compound that binds to DNAJA1

**Fig. 1:**
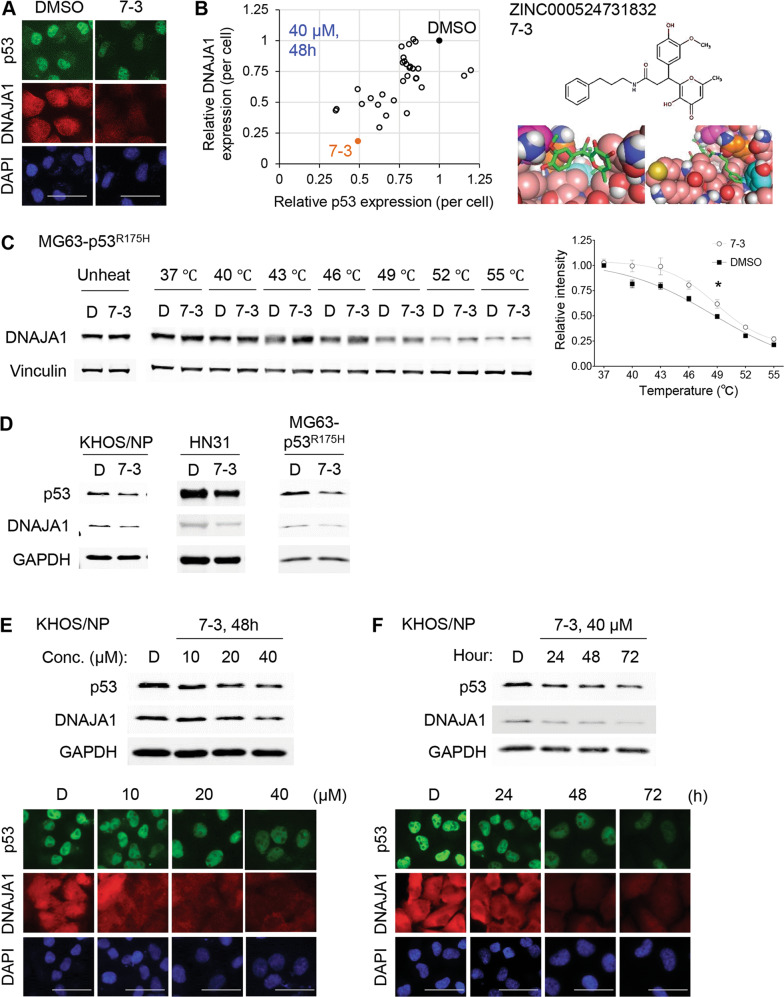
A virtual screen identifies a compound that binds to DNAJA1. **A** Immunofluorescence for p53, DNAJA1, and DAPI, using KHOS/NP (p53^R156P^) cells treated with 7-3 (40 µM, 48 h). Scale bar: 50 µm. **B** Summary of relative immunofluorescence intensities of p53 and DNAJA1, using KHOS/NP cells treated with top 32 compounds (40 µM, 48 h, left). Chemical structure of 7-3 and images from molecular docking studies (right), showing binding of 7-3 with the DNAJA1 J-domain at Y7 (cyan), K44 (magenta), and Q47 (orange). **C** CETSA for DNAJA1 and Vinculin using MG63-p53^R175H^ cells treated with DMSO (D) or 7-3 (100 µM, 4 h) with a quantitative graph. Mean ± SEM (*n* = 4). *p < 0.05; two-way ANOVA. **D** Western blotting for indicated proteins using KHOS/NP, HN31 (p53^C176F^), and MG63-p53^R175H^ cells treated with D or 7-3 (40 µM, 48 h). **E**, **F** Western blotting or immunofluorescence for indicated proteins using KHOS/NP cells treated with different concentrations of D or 7-3 for 48 h (**E**) and at 40 µM of D or 7-3 for 24–72 h (**F**). Scale bar: 50 µm.

DNAJA1 binds to and stabilizes specifically conformational mutp53, while its knockdown induces proteasomal degradation of conformational mutp53 leading to reduced migratory potential [18, 23–25]. Despite the potential of DNAJA1 as a therapeutic target, no specific inhibitor for DNAJA1 or HSP40/JDPs is clinically available. We therefore conducted an in silico docking study using the ZINC database and selected the 32 compounds as described in Supplementary Materials and Methods (Table 1). These 32 compounds were tested for their abilities to deplete DNAJA1 and conformational mutp53 in KHOS/NP (p53^R156P^) cells by immunofluorescence, resulting in identification of a compound “7-3” (Fig. 1A). 7-3 showed the highest activity to reduce both DNAJA1 and p53^R156P^ protein levels (Fig. 1B, left). The virtual screen also identified tyrosine 7 (Y7), lysine 44 (K44), and glutamine 47 (Q47) as predicted amino acids crucial for binding between 7-3 and DNAJA1 (Fig. 1B, right).

**Table 1:** Compounds tested with potential to bind to the J-domain of DNAJA1 through a docking study.

Cluster #	Rank within cluster	Structure	IUPAC	ZINC ID	Score
1	1		1-[(2,6-dimethyl-3-oxo-3,4-dihydro-2H-1,4-benzoxazin-7-yl)sulfonyl]-N-[3-(pyrrolidin-1-yl)propyl]piperidine-4-carboxamide	ZINC000032959058	−62.02
	2		N-[3-(azepan-1-yl)propyl]-1-[(6-methyl-3-oxo-3,4-dihydro-2H-1,4-benzoxazin-7-yl)sulfonyl]piperidine-4-carboxamide	ZINC000032958658	−57.346
	3		N-[2-(azepan-1-yl)ethyl]-1-[(2,6-dimethyl-3-oxo-3,4-dihydro-2H-1,4-benzoxazin-7-yl)sulfonyl]piperidine-3-carboxamide	ZINC000106304218	−49.468
2	1		N-[(furan-2-yl)methyl]-4-[(1E)-3-oxo-3-{4-[2-oxo-2-(pyrrolidin-1-yl)ethyl]piperazin-1-yl}prop-1-en-1-yl]benzene-1-sulfonamide	ZINC000012749184	−56.415
3	1		N-[4-({[(2 R,4 S,5 R)-5-[3-(furan-2-yl)-1-methyl-1H-pyrazol-5-yl]-1-azabicyclo[2.2.2]octan-2-yl]methyl}sulfamoyl)phenyl]acetamide	ZINC000008635407	−52.588
4	1		3-[(4-hydroxypiperidin-1-yl)({1-[(oxolan-2-yl)methyl]-1H-1,2,3,4-tetrazol-5-yl})methyl]-6-methoxy-1,2-dihydroquinolin-2-one	ZINC000057594491	−51.514
5	1		1-[(2,4-dimethoxyphenyl)[1-(2-methoxyethyl)-1H-1,2,3,4-tetrazol-5-yl]methyl]piperidine-4-carboxamide	ZINC000004862266	−51.382
	2		1-[(3,4-dimethoxyphenyl)[1-(2-phenylethyl)-1H-1,2,3,4-tetrazol-5-yl]methyl]-4-(prop-2-en-1-yl)piperazine	ZINC000022929948	−51.382
6	1		3-(2-fluorophenyl)-6,7-dimethoxy-2-methyl-N-[3-(morpholin-4-yl)propyl]-1-oxo-1,2,3,4-tetrahydroisoquinoline-4-carboxamide	ZINC000020114243	−49.446
7	1		3-(4-hydroxy-3-methoxyphenyl)-3-(3-hydroxy-6-methyl-4-oxo-4H-pyran-2-yl)-N-[(1H-indol-4-yl)methyl]propanamide	ZINC000524730478	−48.607
	2		3-(4-hydroxy-3-methoxyphenyl)-3-(3-hydroxy-6-methyl-4-oxo-4H-pyran-2-yl)-N-[2-(1H-indol-1-yl)ethyl]propanamide	ZINC000604405644	−42.833
	3		3-(4-hydroxy-3-methoxyphenyl)-3-(3-hydroxy-6-methyl-4-oxo-4H-pyran-2-yl)-N-(3-phenylpropyl)propanamide	ZINC000524731832	−36.32
11	1		2-(benzylsulfanyl)-5-methyl-7-(3,4,5-trimethoxyphenyl)-4H,7H-[1,2,4]triazolo[1,5-a]pyrimidine-6-carboxamide	ZINC000004176684	−44.804
	2		7-(4-ethoxy-3-methoxyphenyl)-2-(3-hydroxypropyl)-5-methyl-4H,7H-[1,2,4]triazolo[1,5-a]pyrimidine-6-carboxamide	ZINC000004177153	−41.082
	3		7-(3,4-dimethoxyphenyl)-2-(3-hydroxypropyl)-5-methyl-4H,7H-[1,2,4]triazolo[1,5-a]pyrimidine-6-carboxamide	ZINC000013121860	−39.028
12	1		3-(4-methoxybenzenesulfonamido)-4-(piperazin-1-yl)-N-[(pyridin-3-yl)methyl]benzamide	ZINC000033033819	−43.071
	2		N-cyclopentyl-4-(piperazin-1-yl)-3-(4-propanamidobenzenesulfonamido)benzamide	ZINC000033034273	−41.204
	3		N-benzyl-3-(3-methoxybenzenesulfonamido)-N-methyl-4-(piperazin-1-yl)benzamide	ZINC000033033923	−39.444
13	1		1-{2-[3-(2,5-dimethylphenyl)-5-(4-ethoxyphenyl)-4,5-dihydro-1H-pyrazol-1-yl]-2-oxoethyl}piperidine-4-carboxamide	ZINC000036342583	−42.952
14	1		ethyl 1-[8-(benzenesulfonyl)-2H,3H-[1,4]dioxino[2,3-g]quinolin-9-yl]piperidine-4-carboxylate	ZINC000020381010	−42.263
15	1		3-[(3’aS,6’aR)-5-ethyl-2,4’,6’-trioxo-5’-(2-phenylethyl)-1,2,3’,3’a,4’,5’,6’,6’a-octahydro-2’H-spiro[indole-3,1’-pyrrolo[3,4-c]pyrrol]-3’-yl]propanamide	ZINC000015969843	−42.242
	2		3-[(3’aS,6’aR)-5-chloro-2,4’,6’-trioxo-5’-(2-phenylethyl)-1,2,3’,3’a,4’,5’,6’,6’a-octahydro-2’H-spiro[indole-3,1’-pyrrolo[3,4-c]pyrrol]-3’-yl]propanamide	ZINC000015969095	−41.049
	3		3-[(3’aS,6’aR)-5,7-dimethyl-2,4’,6’-trioxo-5’-(2-phenylethyl)-1,2,3’,3’a,4’,5’,6’,6’a-octahydro-2’H-spiro[indole-3,1’-pyrrolo[3,4-c]pyrrol]-3’-yl]propanamide	ZINC000015969934	−37.396
18	1		3-(2-methoxyethyl)-N-[2-(morpholin-4-yl)ethyl]-2,4-dioxo-1,2,3,4-tetrahydroquinazoline-7-carboxamide	ZINC000020117031	−41.207
19	2		1-[3-(diethylamino)propyl]-4-(2,3-dihydro-1,4-benzodioxine-6-carbonyl)-3-hydroxy-5-(3-hydroxyphenyl)-2,5-dihydro-1H-pyrrol-2-one	ZINC000020328954	−38.392
	3		1-[3-(dimethylamino)propyl]-3-hydroxy-5-(4-hydroxy-3-methoxyphenyl)-4-(4-methoxy-2-methylbenzoyl)-2,5-dihydro-1H-pyrrol-2-one	ZINC000017194559	−38.008
21	1		3-(4-chlorophenyl)-N-[2-(3,4-dihydroxyphenyl)ethyl]-3-{2-hydroxy-4-oxo-4H-pyrido[1,2-a]pyrimidin-3-yl}propanamide	ZINC000096231695	−39.604
	2		3-(4-chlorophenyl)-N-[2-(3,4-dihydroxyphenyl)ethyl]-3-{4-hydroxy-2-oxo-2H-pyrido[1,2-a]pyrimidin-3-yl}propanamide	ZINC000206246138	−38.735
22	1		N-[1-(4-benzylpiperazin-1-yl)-3-methyl-1-oxobutan-2-yl]-2-oxo-2,3,4,5-tetrahydro-1H-1-benzazepine-7-sulfonamide	ZINC000020981310	−38.296
23	1		6-[3-(ethoxycarbonyl)piperidin-1-yl]-5-[4-(propan-2-yl)benzenesulfonamido]pyridine-3-carboxylic acid	ZINC000064843663	−37.823
28	1		2-[5-(hydroxymethyl)-1H-indol-3-yl]-2-(4-{2-[(2-oxo-2H-chromen-4-yl)amino]ethyl}piperazin-1-yl)acetic acid	ZINC000031810008	−36.622
29	1		3-(3-hydroxy-4-methoxyphenyl)-3-(4-hydroxy-6-methyl-2-oxo-2H-pyran-3-yl)-N-(3-methoxyphenyl)propanamide	ZINC000253471635	−36.223

To validate the 7-3−DNAJA1 binding, we performed cellular thermal shift assay (CETSA), which is based on the biophysical principle of ligand-induced thermal stabilization of target proteins in live cells [26–29]. Increased DNAJA1 levels were detected in the supernatants of 7-3-treated p53^R175H^-expressing MG63 (p53^null^) cells, as compared to those treated with DMSO, indicating intracellular binding of 7-3 to DNAJA1 (Fig. 1C).

We confirmed that 7-3 reduced protein levels of DNAJA1 and conformational mutp53 in multiple cell lines, including KHOS/NP (p53^R156P^), HN31 (p53^C176F^), and p53^R175H^-expressing MG63 (Fig. 1D). Furthermore, 7-3 depleted DNAJA1 and mutp53 in a concentration- and time-dependent manner (Fig. 1E, F, Supplementary Fig. 1A-D).

### Analogue screen identifies A11, showing an increased activity to deplete DNAJA1 and conformational mutp53

**Fig. 2:**
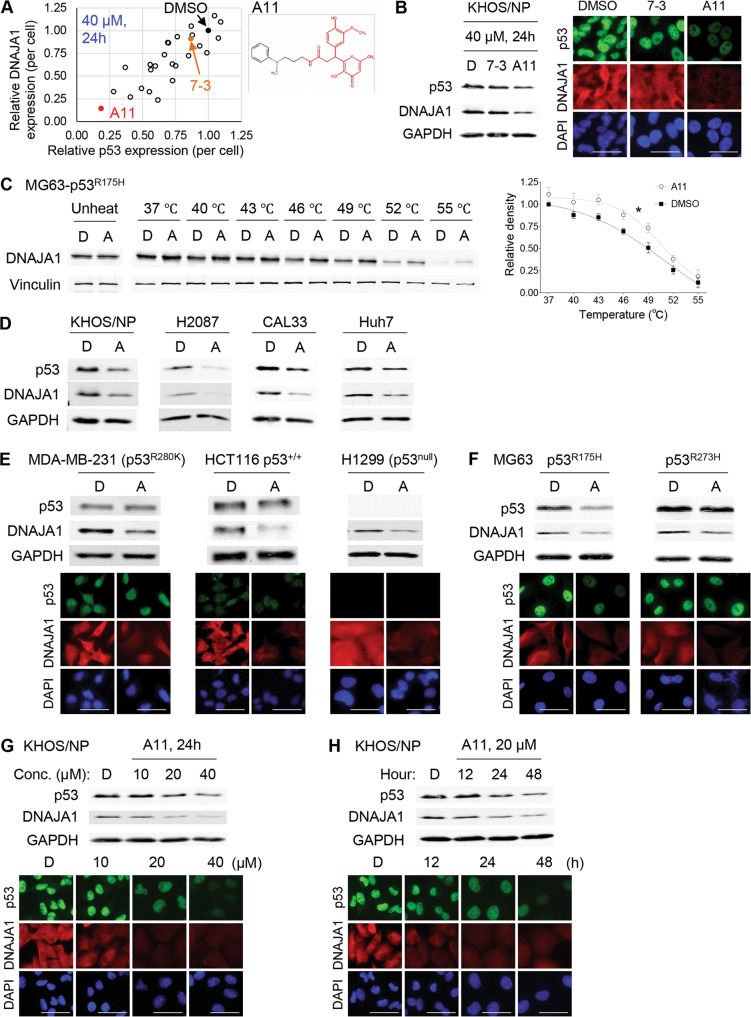
Analogue screen identifies A11, showing an increased activity to deplete DNAJA1 and conformational mutp53. **A** Summary of relative immunofluorescence intensity for DNAJA1 and p53 using KHOS/NP cells treated with 7-3 and 25 analogues (40 µM, 24 h) and chemical structure of A11. **B** Western blotting and immunofluorescence for indicated proteins using KHOS/NP cells treated with DMSO (D), 7-3, or A11 (40 µM, 24 h). Scale bar: 50 µm. **C** CETSA for DNAJA1 and Vinculin using MG63-p53^R175H^ cells treated with D or A11 (A, 100 µM, 4 h) with a quantitative graph. Mean ± SEM (*n* = 4). *p < 0.05; two-way ANOVA. **D** Western blotting for indicated proteins using KHOS/NP (p53^R156P^), H2087 (p53^V157F^), CAL33 (p53^R175H^), and Huh7 (p53^Y220C^) cells treated with D or A (20 µM, 24 h). **E**, **F** Western blotting and immunofluorescence for indicated proteins, using MDA-MB-231 (p53^R280K^), HCT116 (p53^+/+^), and H1299 (p53^null^) cells (**E**), as well as MG63-p53^R175H^ and MG63-p53^R273H^ cells (F), treated with A11 (20 µM, 24 h). Scale bar: 50 µm. **G**, **H** Western blotting or immunofluorescence for indicated proteins using KHOS/NP cells treated with different concentrations of D or A11 for 24 h (**G**) and at 20 µM of D or A11 for 12–48 h (H). Scale bar: 50 µm.

7-3 decreased the protein levels of both DNAJA1 and conformational mutp53, which required high centration (40 µM) and a long-time treatment period (48 h). Hence, we selected 25 analogues based on a search of the ChemSpider (http://www.chemspider.com/) (Table 2). These compounds were tested for their abilities to reduce DNAJA1 and p53^R156P^ levels in KHOS/NP cells for a shorter treatment period (24 h) at 40 µM (Fig. 2A). A compound “A11” showed the highest activity to reduce these proteins (Fig. 2A, B). CETSA confirmed the binding of A11 to DNAJA1 in p53^R175H^-expressing MG63 cells (Fig. 2C). A11 depleted DNAJA1 and conformational mutp53 in multiple cell lines, including KHOS/NP (p53^R156P^), H2087 (p53^V157F^), CAL33 (p53^R175H^), and Huh7 (p53^Y220C^) cells, at lower concertation (20 µM) and a shorter treatment period (24 h) than those used for 7-3 (Fig. 2D).

**Table 2:** Analogues of 7-3 tested.

Compound name	Structure	IUPAC
7-5		N-(1,1-dioxo-1λ^6^-thiolan-3-yl)-3-(3-hydroxy-6-methyl-4-oxo-4H-pyran-2-yl)-3-(4-methoxyphenyl)propanamide
7-6		N-[2-(dimethylamino)-2-(furan-2-yl)ethyl]-3-(4-hydroxy-3-methoxyphenyl)-3-(3-hydroxy-6-methyl-4-oxo-4H-pyran-2-yl)propanamide
7-7		N-[3-(1H-1,3-benzodiazol-2-yl)propyl]-3-(3-hydroxy-6-methyl-4-oxo-4H-pyran-2-yl)-3-(4-methoxyphenyl)propanamide
7-8		N-[3-(furan-2-yl)propyl]-3-(4-hydroxy-3-methoxyphenyl)-3-(3-hydroxy-6-methyl-4-oxo-4H-pyran-2-yl)propanamide
7-9		N-{[4-(dimethylamino)phenyl]methyl}-3-(4-hydroxy-3-methoxyphenyl)-3-(3-hydroxy-6-methyl-4-oxo-4H-pyran-2-yl)propanamide
7-10		3-(4-hydroxy-3-methoxyphenyl)-3-(3-hydroxy-6-methyl-4-oxo-4H-pyran-2-yl)-N-[(1H-indol-4-yl)methyl]propanamide
A1		3-(4-hydroxy-3-methoxyphenyl)-3-(3-hydroxy-6-methyl-4-oxo-4H-pyran-2-yl)-N-(2-phenoxyethyl)propanamide
A2		N-[(1 S)-1-(1H-1,3-benzodiazol-2-yl)ethyl]-3-(4-hydroxy-3-methoxyphenyl)-3-(3-hydroxy-6-methyl-4-oxo-4H-pyran-2-yl)propanamide
A3		N-[3-(1H-1,3-benzodiazol-2-yl)propyl]-3-(4-hydroxy-3-methoxyphenyl)-3-(3-hydroxy-6-methyl-4-oxo-4H-pyran-2-yl)propanamide
A4		3-(4-hydroxy-3-methoxyphenyl)-3-(3-hydroxy-6-methyl-4-oxo-4H-pyran-2-yl)-N-[3-(5-methoxy-1H-indol-1-yl)propyl]propanamide
A5		3-(4-hydroxy-3-methoxyphenyl)-3-(3-hydroxy-6-methyl-4-oxo-4H-pyran-2-yl)-N-(2-{imidazo[1,2-a]pyridin-2-yl}ethyl)propanamide
A6		3-(4-hydroxy-3-methoxyphenyl)-3-(3-hydroxy-6-methyl-4-oxo-4H-pyran-2-yl)-N-(3-{[1,2,4]triazolo[4,3-a]pyridin-3-yl}propyl)propanamide
A7		3-(4-hydroxy-3-methoxyphenyl)-3-(3-hydroxy-6-methyl-4-oxo-4H-pyran-2-yl)-N-{3-oxo-3-[2-(pyridin-3-yl)piperidin-1-yl]propyl}propanamide
A8		3-(4-hydroxy-3-methoxyphenyl)-3-(3-hydroxy-6-methyl-4-oxo-4H-pyran-2-yl)-N-(2-{[(1 S,9 S)-6-oxo-7,11-diazatricyclo[7.3.1.0^2^,^7^]trideca-2,4-dien-11-yl]sulfonyl}ethyl)propanamide
A9		N-(3,3-diphenylpropyl)-3-(4-hydroxy-3-methoxyphenyl)-3-(3-hydroxy-6-methyl-4-oxo-4H-pyran-2-yl)propanamide
A10		N-(3,3-diphenylpropyl)-3-(4-hydroxy-3-methoxyphenyl)-3-(3-hydroxy-6-methyl-4-oxo-4H-pyran-2-yl)propanamide
A11		3-(4-hydroxy-3-methoxyphenyl)-3-(3-hydroxy-6-methyl-4-oxo-4H-pyran-2-yl)-N-{3-[methyl(phenyl)amino]propyl}propanamide
A12		3-(3-hydroxy-6-methyl-4-oxo-4H-pyran-2-yl)-3-(4-methoxyphenyl)-N-[2-oxo-2-(1,2,3,4-tetrahydroisoquinolin-2-yl)ethyl]propanamide
B1		3-(4-hydroxy-3-methoxyphenyl)-3-(3-hydroxy-6-methyl-4-oxo-4H-pyran-2-yl)-N-{2-[2-(pyridin-3-yl)-1,3-thiazol-4-yl]ethyl}propanamide
B2		3-(4-hydroxy-3-methoxyphenyl)-3-(3-hydroxy-6-methyl-4-oxo-4H-pyran-2-yl)-N-{2-[(pyridin-2-yl)carbamoyl]ethyl}propanamide
B3		3-(4-hydroxy-3-methoxyphenyl)-3-(3-hydroxy-6-methyl-4-oxo-4H-pyran-2-yl)-N-{2-[3-(pyridin-2-yl)-1,2,4-oxadiazol-5-yl]ethyl}propanamide
B4		(2 S)-N-[(1,3-dimethyl-2,6-dioxo-1,2,3,6-tetrahydropyrimidin-4-yl)methyl]-2-[3-(4-hydroxy-3-methoxyphenyl)-3-(3-hydroxy-6-methyl-4-oxo-4H-pyran-2-yl)propanamido]propanamide
B5		N-[(1,3-dimethyl-2,6-dioxo-1,2,3,6-tetrahydropyrimidin-4-yl)methyl]-3-[3-(4-hydroxy-3-methoxyphenyl)-3-(3-hydroxy-6-methyl-4-oxo-4H-pyran-2-yl)propanamido]propanamide
B6		3-(4-hydroxy-3-methoxyphenyl)-3-(3-hydroxy-6-methyl-4-oxo-4H-pyran-2-yl)-N-{[(pyridin-2-yl)carbamoyl]methyl}propanamide
C1		4-{2-[3-(4-hydroxy-3-methoxyphenyl)-3-(3-hydroxy-6-methyl-4-oxo-4H-pyran-2-yl)propanamido]acetamido}benzamide

Next, we treated multiple cell lines expressing DNA-contact mutp53 (p53^R248L^, p53^R248Q^, p53^R273H^, p53^R280K^), wtp53, or p53 null with A11 at 20 µM for 24 h. A11 showed little effect on DNA-contact mutp53 and wtp53, although it reduced DNAJA1 levels (Fig. 2E, Supplementary Fig. 2A). Consistently, using MG63 cells exogenously expressing p53^R175H^ or p53^R273H^, we confirmed that A11 depleted p53^R175H^, but not p53^R273H^ (Fig. 2F), showing the specificity of A11 on conformational mutp53.

We also found that A11 reduced DNAJA1 protein levels at a concentration as low as 10 µM and as early as 12 h post-treatment, while it depleted conformational mutp53 at as low as 20 µM and as early as 24 h post-treatment, in a concentration- and treatment period-dependent manner in KHOS/NP, CAL33, and p53^R175H^-expressing MG63 cells (Fig. 2G, H, Supplementary Fig. 2B-E).

### A11 induces proteasomal degradation of DNAJA1 and subsequently reduces conformational mutp53

**Fig. 3:**
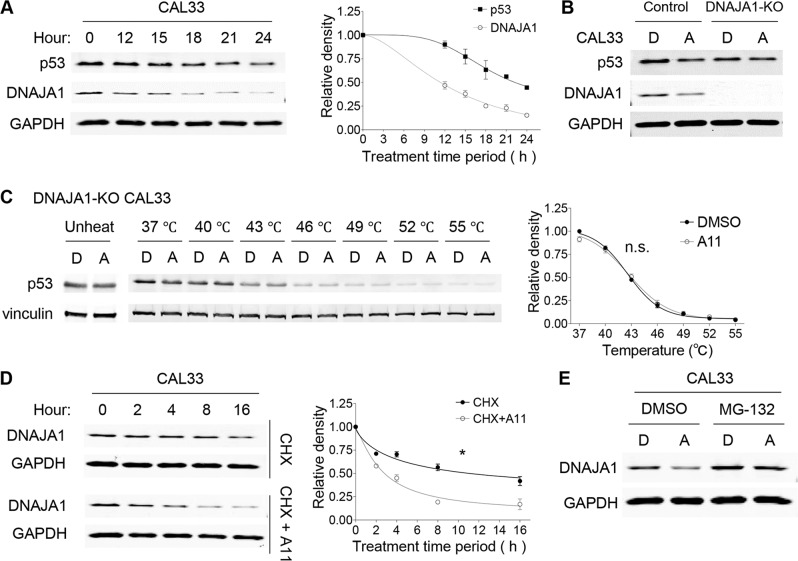
A11 induces proteasomal degradation of DNAJA1 and subsequently reduces conformational mutp53. **A** Western blotting for indicated proteins using CAL33 cells treated with A11 at 20 µM for 12–24 h with a quantitative graph. Mean ± SEM (*n* = 3). **B** Western blotting for indicated proteins using control and DNAJA1-KO CAL33 cells treated with DMSO (D) or A11 (A, 20 µM, 24 h). **C** CETSA for p53 and Vinculin with DNAJA1-KO CAL33 cells treated with D or A (100 µM, 4 h) with a quantitative graph. Mean ± SEM (*n* = 3). n.s. not significant; two-way ANOVA. **D** Western blotting for indicated proteins using CAL33 cells treated with A11 at 40 µM with or without cycloheximide (CHX, 100 ng/mL) for different treatment periods with a quantitative graph. Mean ± SEM (*n* = 3). *p < 0.05; two-way ANOVA. **E** Western blotting for DNAJA1 and GAPDH using CAL33 cells treated with D or A (20 µM, 12 h) and additional D or A treatments with or without MG-132 (30 µM, 8 h).

To examine whether depletion of DNAJA1 preceded to that of conformational mutp53, we performed kinetic studies of DNAJA1 and mutp53 in CAL33 and KHOS/NP cells between 12 h and 24 h following 20 µM of A11 treatment. A11 decreased DNAJA1 levels earlier than mutp53 in both cells (Fig. 3A, Supplementary Fig. 3A). Moreover, A11 had little effect on the protein levels of mutp53 in DNAJA1-knockout (DNAJA1-KO) CAL33 and KHOS/NP cells (Fig. 3B, Supplementary Fig. 3B). CETSA revealed lack of binding of A11 to conformational mutp53 in DNAJA1-KO CAL33 cells (Fig. 3C). A11 also failed to interfere with the DNAJA1−mutp53 binding by co-immunoprecipitation studies (Supplementary Fig. 3C). These results suggest that A11-mediated depletion of mutp53 is dependent on the presence of DNAJA1.

Additionally, we confirmed that A11 did not affect mRNA levels of *DNAJA1* and *p53* in KHOS/NP and Huh7 cells (Supplementary Fig. 3D, E). Also, A11-mediated depletion of mutp53 was not resultant of reactivation or conformational changes of mutp53 to wtp53 with a shorter half-life; A11 did not increase mRNA expression of p53-downstream target genes (Supplementary Fig. 3F, G). These observations suggest that A11 regulates protein levels of DNAJA1 and conformational mutp53 at the post-transcriptional level.

We hence determined the effects of A11 on the protein half-life of DNAJA1 in CAL33. A11 significantly shortened the DNAJA1 protein half-life from 11.6 to 3.2 h (Fig. 3D), suggesting reduction of DNAJA1 protein stability by A11. Indeed, a proteasome inhibitor substantially rescued A11-mediated reduction in the DNAJA1 protein level (Fig. 3E). Thus, A11 binds to and induces proteasomal degradation of DNAJA1, which triggers degradation of conformational mutp53.

### A11 inhibits migratory potential in a manner dependent on DNAJA1 and conformational mutp53

**Fig. 4:**
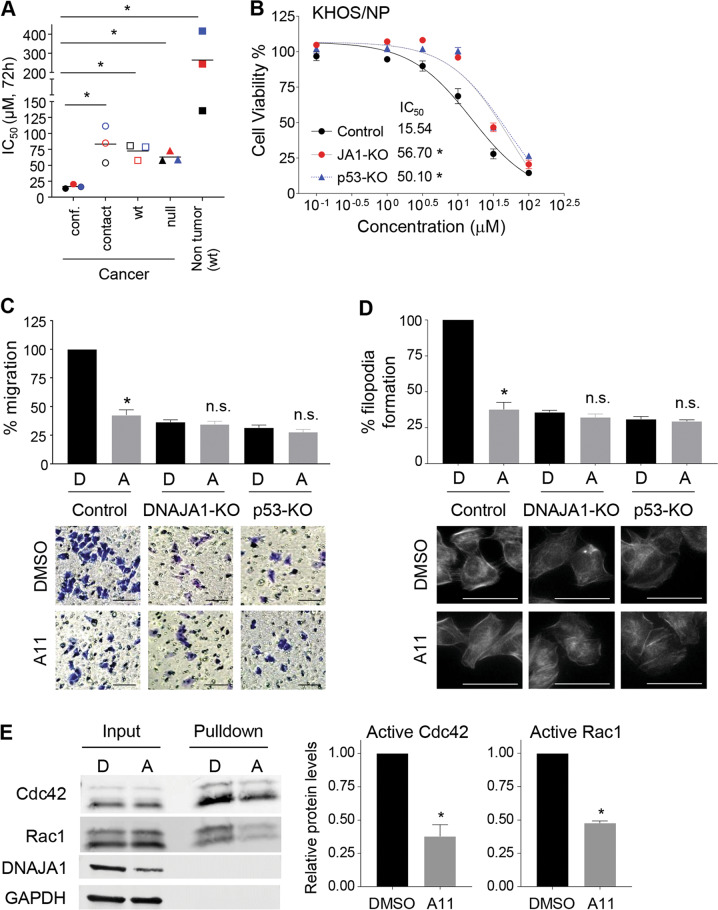
A11 inhibits migratory potential in a manner dependent on DNAJA1 and conformational mutp53. **A** Summary of 72h-IC_50_ values of A11 in multiple human cancer and non-tumor cell lines. 72h-IC_50_s (µM) for cancer cells with conformational mutp53 (conf.): CAL33 (black, 13.8 µM), Huh7 (red, 20.7), KHOS/NP (blue, 16.6); DNA-contact mutp53 (contact): HSC4 (black, 53.7), FaDu (red, 84.8), MDA-MB-231 (blue, 111.4); wild-type p53 (wt): U2OS (black, 80.7), SJSA1 (red, 57.7), HCT116 p53^+/+^ (blue, 78.6); p53 null (null): H1299 (black, 57.9), MG63 (red, 72.6), HCT116 p53^−/−^ (blue, 58.7). Non-tumor cells (wtp53): HOE (black, 136.1), WI-38 (red, 243.4), BJ (blue, 414.9). *p < 0.05; two-tailed Student’s *t*-test (*n* = 3). **B** MTT assays (Mean ± SEM, *n* = 8), using control, *DNAJA1*-knockout (JA1-KO), and *p53*-knockout (p53-KO) KHOS/NP cells. **C** Summary and images of transwell migration assays using control, DNAJA1-KO, or p53-KO KHOS/NP cells treated with DMSO (D) or A11 (A, 20 µM, 12 h). Cells were pre-treated with A11 for 12 h. *p < 0.05; two-tailed Student’s *t*-test (*n* = 3). n.s. not significant. Scale bar: 100 µm. **D** Summary and images of F-actin staining using indicated KHOS/NP subcell lines treated with A11 (20 µM, 24 h). *p < 0.05; two-tailed Student’s *t*-test (*n* = 3). n.s. not significant. Scale bar: 50 µm. **E** Blots and summary of Rac1/Cdc42 activation assays using KHOS/NP cells treated with D or A (20 µM, 18 h). Mean ± SEM (*n* = 3). *p < 0.05; two-tailed Student’s *t*-test.

Given frequent addiction of cancer cells to mutp53, we hypothesized that cells expressing conformational mutp53 are sensitive to A11. We determine 72h-IC_50_ values for A11 by MTT assays in multiple cancer cell lines with different p53 status and wtp53-expressing non-tumor cell lines. Cancer cells harboring conformational mutp53 showed significantly lower 72h-IC_50_ values as compared to those with other p53 status (DNA-contact, wtp53, p53 null) and non-tumor cells (Fig. 4A). To furthermore examine the dependency of A11’s activity to inhibit viable cell proliferation on DNAJA1 and conformational mutp53, we used DNAJA1-knockout (JA1-KO) and mutp53-knockout (p53-KO) KHOS/NP and CAL33 cells. Although JA1-KO and p53-KO cells showed decreased sensitivity to A11 as compared to the control cells, A11 still inhibited cell viability of these cells (Fig. 4B, Supplementary Fig. 4A), suggesting DNAJA1-independent activities of A11.

Since migratory potential is one of the most prominent mutp53 GOF activities, we examined the effects of A11 on suppressing migratory potential of control, DNAJA1-KO, and p53-KO KHOS/NP cells by transwell migration assays. A11 significantly inhibited migration of control cells, with little effect on migration of DNAJA1- or mutp53-lacking cells (Fig. 4C).

DNAJA1 regulates filopodia-forming potential dependent on mutp53, which contributes to cell migration [18]. Hence, we performed F-actin staining using the KHOS/NP isogenic cell lines. Expectedly, A11 failed to further inhibit filopodia formation of cells lacking DNAJA1 or mutp53 (Fig. 4D). To support these findings, A11 showed minimal effects on the migratory- and filopodia-forming potential of cancer cells expressing DNA-contact mutp53 (MDA-MB-231: p53^R280K^, FaDu: p53^R248L^, HSC4: p53^R248Q^), wtp53 (SJSA1, U2OS, HCT116 p53^+/+^), and p53 null (MG63, HCT116 p53^−/−^, H1299) (Supplementary Fig. 4B, C).

Activation of Cdc42 and Rac1 is crucial for enhancing filopodia formation [18]. Indeed, A11 reduced active forms of Cdc42 and Rac1 in KHOS/NP cells (Fig. 4E). Together, the effects of A11 on inhibiting filopodia formation and migration are dependent on DNAJA1 and conformational mutp53.

### A11 decreases protein levels of other HSP40/JDP members

**Fig. 5:**
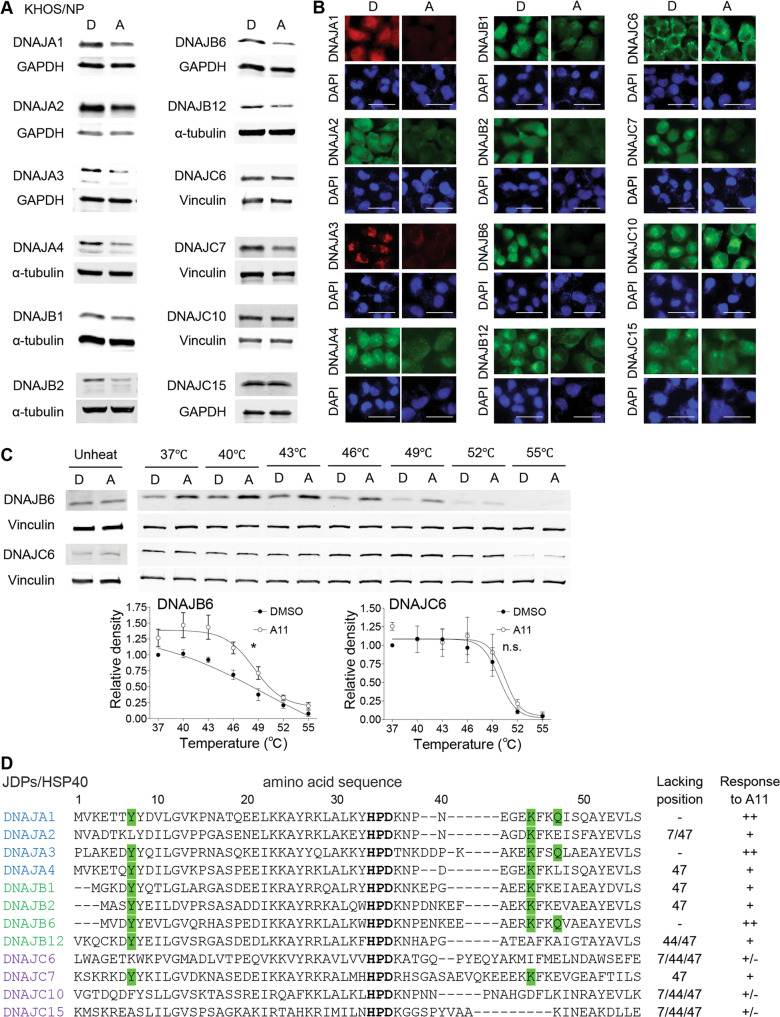
A11 decreases protein levels of other HSP40/JDP members. **A**, **B** Western blotting (**A**) and immunofluorescence (**B**) for indicated proteins, using KHOS/NP cells treated with DMSO (D) or A11 (A, 20 µM, 24 h). Scale bar: 50 µm. **C** CETSA for DNAJB6, DNAJC6, and Vinculin, using MG63-p53^R175H^ cells treated with D or A. A quantitative graph (lower). Mean ± SEM (*n* = 3). *p < 0.05; two-way ANOVA. n.s. not significant. **D** Amino acid sequence alignment of J-domains of HSP40/JDPs, with information of positions at Y7, K44, Q47 (green) and response to A11 (Good: ++, some response: +, little or no: +/−).

A11 appears to have both DNAJA1-dependent (migratory potential) and -independent (viable proliferation) activities. Considering the well-conserved J-domains among HSP40/JDPs, we tested the hypothesis that A11 could bind to and reduce protein levels of other HSP40/JDPs. We tested multiple HSP40/JDP members, including DNAJA1, DNAJA2, DNAJA3, DNAJA4, DNAJB1, DNAJB2, DNAJB6, DNAJB12, DNAJC6, DNAJC7, DNAJC10, and DNAJC15. In both KHOS/NP and CAL33 cells, A11 consistently depleted DNAJA1, DNAJA3, and DNAJB6, while it failed to reduce levels of DNAJC6, DNAJC10, and DNAJC15 (Fig. 5A, B, Supplementary Fig. 5A). Other HSP40/JDP members showed varying degrees of response to A11.

To exclude the possibility that reduction in DNAJA3 and DNAJB6 levels could be secondary to depletion of DNAJA1 or mutp53, we compared protein levels of DNAJA3 and DNAJB6, as well as DNAJC6 and DNAJC10 as negative controls, among control, DNAJA1-KO, and p53-KO CAL33 cells. There was no difference in these protein levels among these subcell lines (Supplementary Fig. 5B), suggesting direct effects of A11 on these protein levels. Indeed, CETSA revealed that A11 bound to DNAJB6, but not DNAJC6, in MG63-p53^R175H^ cells (Fig. 5C).

Next, we examined possible correlation between A11 response and amino acid sequences of J-domains among HSP40/JDP members examined above (Fig. 5D). Good responders (DNAJA1, DNAJA3, DNAJB6) had three predicted amino acids (Y7, K44, Q47) key for the 7-3−DNAJA1 binding (Fig. 1B), while little or no responders (DNAJC6, DNAJC10, DNAJC15) lacked all these three residues (Fig. 5D). These results may suggest critical roles of these three residues in A11’s binding to HSP40/JDPs and subsequent depletion of the binding targets.

### A triple mutant DNAJA1 with alanine mutations at Y7, K44, and Q47 does not respond to A11

**Fig. 6:**
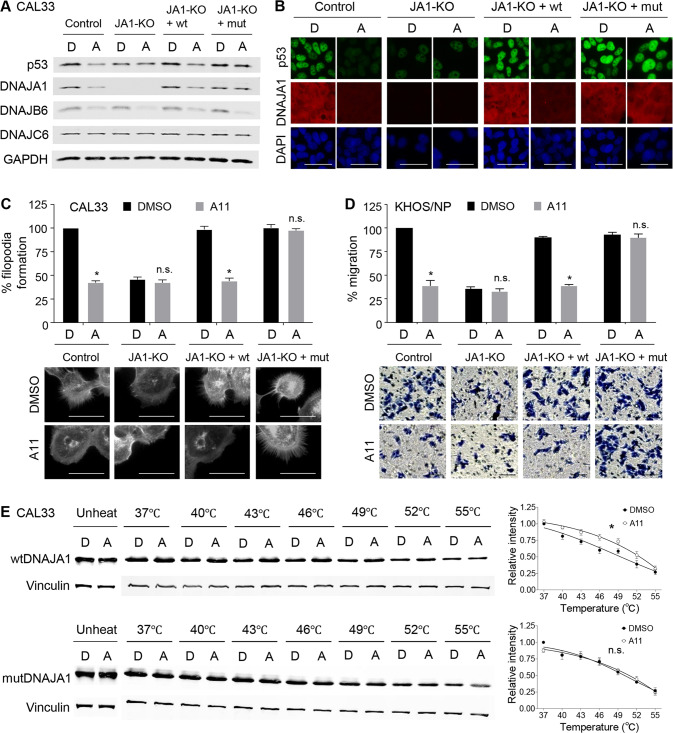
A triple mutant DNAJA1 does not respond to A11. **A**, **B** Western blotting (**A**) and immunofluorescence (**B**) for indicated proteins, using control and JA1-KO CAL33 cells with or without wtDNAJA1 (wt) or triple mutDNAJA1 (mut), treated with DMSO (D) or A11 (A, 20 µM, 24 h). Scale bar: 50 µm. **C** Summary and images of F-actin staining using CAL33 subcell lines treated with D or A (20 µM, 24 h). Scale bar: 50 µm. Mean ± SEM (*n* = 3). *p < 0.05; two-tailed Student’s *t*-test. n.s. not significant. **D** Summary and images of transwell migration assays, using KHOS/NP subcell lines treated with D or A (20 µM, 12 h). Cells were pre-treated with D or A for 12 h. Mean ± SEM (*n* = 3). *p < 0.05; two-tailed Student’s *t*-test. n.s. not significant. Scale bar: 100 µm. **E** CETSA for wtDNAJA1, mutDNAJA1, and Vinculin, using wtDNAJA1 or mutDNAJA1-expressing JA1-KO CAL33 cells treated with D or A, with quantitative graphs. Mean ± SEM (*n* = 4). *p < 0.05; two-way ANOVA. n.s. not significant.

To examine the significance of Y7, K44, and Q47 residues on A11-mediated cellular phenotypes, we generated a triple mutant DNAJA1 with alanine substitution (Y7A/K44A/Q47A: mutDNAJA1), followed by re-introduction of wild-type DNAJA1 (wtDNAJA1) or mutDNAJA1 into DNAJA1-KO CAL33 cells. First, we compared protein levels of mutp53 (p53^R175H^) and DNAJA1 and their response to A11 between wtDNAJA1- and mutDNAJA1-expressing DNAJA1-KO CAL33 cells (Fig. 6A, B). The levels of mutp53 in DNAJA1-KO cells were restored by wtDNAJA1 and mutDNAJA1. A11 efficiently reduced mutp53 levels in cells with wtDNAJA1, whereas it failed to reduce mutp53 in cells expressing mutDNAJA1. We also confirmed that A11 reduced endogenous DNAJB6, but not DNAJC6, in all subcell lines examined including DNAJA1-KO CAL33 cells. Essentially, the same results were obtained using DNAJA1-KO KHOS/NP cells re-introduced for wtDNAJA1 or mutDNAJA1 (Supplementary Fig. 6A, B).

Next, we examined the effects of A11 on filopodia formation and migration using these CAL33 and KHOS/NP subcell lines (Fig. 6C, D). Both wtDNAJA1 and mutDNAJA1 rescued the filopodia-forming and migratory potential of DNAJA1-KO CAL33 and KHOS/NP cells, respectively. A11 reduced filopodia formation and migration in cells expressing either endogenous (control) or exogenous wtDNAJA1; however, DNAJA1-KO cells and those expressing mutDNAJA1 failed to respond to A11. Furthermore, we confirmed that A11 bound to exogenously expressed wtDNAJA1, but not mutDNAJA1, in DNAJA1-KO CAL33 cells by CETSA (Fig. 6E). Together, these results indicate that binding of A11 to DNAJA1 through Y7, K44, and Q47 is crucial for triggering degradation of DNAJA1 and subsequent depletion of conformational mutp53, leading to inhibition of filopodia formation and migration.

## Discussion

This is the first study identifying an uncharacterized DNAJA1 inhibitor through a virtual screen of ~10 million compounds for the J-domain of DNAJA1 using a protocol designed by Johnson et al [30, 31]. We identify “A11” that depletes DNAJA1 and subsequently conformational mutp53. A11 inhibits cancer cell migration in a manner dependent on DNAJA1 and conformational mutp53, showing the on-target effect. A11’s activity appears to be dependent on Y7, K44, and Q47 in J-domain. Indeed, A11 binds to and reduces protein levels of other HSP40/JDPs containing these three amino acids. Since each HSP40/JDP likely has different client proteins, this could explain the DNAJA1- or conformational mutp53-independent anti-proliferative activity of A11.

Moses et al. [32] previously identified chalcone C86 that induces degradation of androgen receptor (AR) and its variant ARv. C86 binds to several members of HSP40/JDPs, including DNAJA1; however, the effects of C86 on mutp53 are not investigated. It is also unclear whether biological effects of C86 are dependent on C86-binding HSP40/JDPs, since cells lacking HSP40/JDPs are not used. Tong et al. [33] recently identified a compound, GY1-22, that interacted with interface of the interacting pocket of DNAJA1 and p53^R175H^ (mouse p53^R172H^), leading to depletion of p53^R175H^. GY1-22 inhibits binding between DNAJA1 and p53^R175H^ in cells, which depletes p53^R175H^ and cyclin D1 with increased p21 levels for unclear reasons and inhibits in vivo tumor growth of p53^R172H^-expressing murine pancreatic carcinoma cells [33]. This study also does not address whether biological phenotypes induced by GY1-22 are dependent on DNAJA1 or p53^R175H^ and if GY1-22 could deplete other p53 mutants than p53^R175H^. Notably, neither C86 nor GY1-22 alters protein levels of DNAJA1, unlike A11. Thus, the mechanism of action of A11 is unique and distinct.

DNAJA1 is also shown to interact with other proteins than mutp53, as a molecular chaperone. DNAJA1 enhances formation of aggregation of polyQ74htt in the Huntington’s disease model [34, 35], reduces aggregation of neurodegenerative disorder-associated tau [36], and promotes folding of newly synthesized cystic fibrosis transmembrane conductance regulator at the endoplasmic reticulum [37–39]. DNAJA1 also stabilizes cell division cycle 45 to promote tumor progression [40], while it binds to transglutaminase 2 associated with cell survival [41]. Thus, A11 could have an impact on these diverse cellular activities and their related disease progression. Future pre-clinical studies, including structural activity relationship to improve efficacy and specificity, as well as the pharmacological and toxicological characterization, are required to identify clinically applicable HSP40/JDP inhibitors.

## Materials and methods

Detailed materials and methods are described in the Supplementary information.

## Supplementary information

Supplemental information

Supplementary Figures

Supplementary table 1

Original Data File

Authors list

## Data Availability

All data are available in the main text or the supplementary materials.
