# Supplementary material for: DNAJA1- and conformational mutant p53-dependent inhibition of cancer cell migration by a novel compound identified through a virtual screen
Source: Cell Death Discov. 2022 Oct 31;8:437. doi: 10.1038/s41420-022-01229-5 (PMC9622836; doi:10.1038/s41420-022-01229-5)
Supplement: Supplementary file 1 — Supplemental information [file 41420_2022_1229_MOESM1_ESM.pdf]

Supplemental information

Supplementary Materials and Methods

Supplementary References

Supplementary Figure Legends

Supplementary Materials and Methods

Virtual screen to identify compounds binding to DNAJA1

A library of around 10 million commercially available compounds was downloaded from ZINC [1]. The library was subjected to two medicinal chemistry filters, with Pan-assay interference compounds (PAINS) [2] as well as compounds flagged by the Eli Lilly Medicinal Chemistry Rules [3] retained, but clearly labeled. Compounds were ionized based on a pH of 7 with FIXPKA (https://www.eyesopen.com/), and then up to 300 conformers were generated using OMEGA (https://www.eyesopen.com/omega) to generate multi-conformer structure databases [4, 5]. Two nuclear magnetic resonance (NMR) ensembles of the J domain of DNAJA1 were available in the PDB, 2LO1 and 2M6Y [6], with each entry containing 20 models within the ensemble. His tags were removed, and proteins were renumbered, when necessary. For each model, an exemplar was generated that was touching both residues G13 and Y34, which is essentially a shape-based pharmacophore of the binding site near the crook of the J domain [7]. The ~10 million compounds were screened using the exemplar screening developed by Johnson et al. [8], with a virtual screening tool ROCS (https://www.eyesopen.com/rocs) [9], to compare the shape and chemical features of the screening library versus the exemplar (binding pocket). Of the top 1% of hits for each input structure, the ROCS overlay scores were normalized, and those with a Z score greater than 1 were subjected to full atom minimization using Rosetta, resulting in 45,735 compound-receptor pairs total [10]. Minimized compounds with more than 2 buried unsatisfied hydrogen bond donors or acceptors at the interface were filtered out, and the remaining top scoring compounds (Rosetta energy of the minimized complex

minus the sum of the minimized energies of unbound protein and free ligand) were selected based on visual inspection of the complex, physiochemical properties, and medicinal chemistry filters. All of the compounds selected for testing passed the PAINS filters.

Chemicals and compounds

Compounds (4-1, 5-1, and 5-2) were obtained from Asinex Corp. (Winston-Salem, NC, US), while all other compounds were purchased from Molport (Latvia). Detailed information containing compound names, suppliers, and catalog numbers is available in Supplementary Table 1.

Cell lines

All the human tumor cell lines (KHOS/NP, HN31, MG63, H2087, CAL33, Huh7, MDA-MB-231, FaDu, HSC4, HCT116, SJSA1, U2OS, H1299) and non-tumor cell lines (WI-38, BJ) were maintained in Dulbecco’s modified Eagle’s medium (DMEM) containing 100 units/ml penicillin and 100 µg/ml streptomycin, supplemented with 10% fetal bovine serum (FBS). Human oral epithelial (HOE) cells were purchased from Celprogen (Torrance, CA, USA) and maintained in HOE media with serum. All cell lines were incubated in a humidified incubator at 37°C with 5% CO_2. All cell lines were authenticated in the University of Arizona Genetics Core facility (Tucson, AZ, USA).

Antibodies

The following antibodies were used for western blotting: mouse monoclonal anti-p53 (sc-126, DO-1, Santa Cruz Biotechnology), rabbit monoclonal anti-p53 (#2527, 7F5, Cell Signaling Technology), goat polyclonal anti-p53 (AF1355, R&D systems), rabbit polyclonal anti-DNAJA1 (HPA001306, Sigma-Aldrich), mouse monoclonal anti-HDJ2 (MA5-12748, KA2A5.6, Invitrogen), rabbit polyclonal anti-GAPDH (10494-1-AP, Proteintech), mouse monoclonal anti-vinculin (05-386, V284, EMD Millipore), mouse monoclonal anti-RAC1 (66122-1-Ig, 4A4B11, Proteintech), rabbit polyclonal anti-CDC42 (ab64533, Abcam), rabbit polyclonal anti-DNAJA2 (12236-1-AP, Proteintech), mouse monoclonal anti-DNAJA3 (sc-18820, RS-11, Santa Cruz Biotechnology), mouse monoclonal anti-DNAJA4 (sc-100714, LL2, Santa Cruz Biotechnology), rabbit polyclonal anti-DNAJA4 (HPA041790, MilliporeSigma), rabbit polyclonal anti-DNAJB1 (13174-1-AP, Proteintech), rabbit polyclonal anti-DNAJB2 (10838-1-AP, Proteintech), rabbit polyclonal anti-DNAJB6 (11707-1-AP, Proteintech), rabbit polyclonal anti-DNAJB12 (6780-1-AP, Proteintech), rabbit polyclonal anti-DNAJC6 (1941-1-AP, Proteintech), rabbit polyclonal anti-DNAJC7 (11090-1-AP, Proteintech), rabbit polyclonal anti-DNAJC10 (13101-1-AP, Proteintech), rabbit polyclonal anti-DNAJC15 (16063-1-AP, Proteintech), mouse monoclonal anti-Alpha Tubulin (66031-1-Ig, Proteintech), IRDye® 680RD Donkey anti-Rabbit IgG (H + L) (926-68073, LI-COR), IRDye® 800CW Donkey anti-Mouse IgG (H + L) (926-32212, LI-COR), and Donkey-anti-goat 550 (AC2164, Azure Biosystems).

Mouse monoclonal anti-HDJ2 antibody (KA2A5.6) was used for immunoprecipitation, while mouse monoclonal anti-GFP antibody (sc-9996, B-2, Santa

Cruz Biotechnology) was used as an isotype control. Goat polyclonal anti-p53 and rabbit polyclonal anti-DNAJA1 were used for immunoprecipitation studies.

The following antibodies were used for immunofluorescence; anti-p53 (DO-1), anti-DNAJA1 (HPA001306), anti-DNAJA2 (12236-1-AP), anti-DNAJA3 (RS-11), anti-DNAJA4 (HPA041790), anti-DNAJB1 (13174-1-AP), anti-DNAJB2 (10838-1-AP), anti-DNAJB6 (11707-1-AP), anti-DNAJB12 (6780-1-AP), anti-DNAJC6 (1941-1-AP), anti-DNAJC7 (11090-1-AP), anti-DNAJC10 (13101-1-AP), and anti-DNAJC15 (16063-1-AP).

Plasmids

The *pBABEpuro-p53*-based retroviral vectors encoding $p53^{R175H}$ and $p53^{R273H}$ were used for overexpression of p53. *DNAJA1* cDNA (GenBank accession number EU176556) was amplified by PCR and inserted into BamHI and NotI restriction sites of the *pCDH-CMV-MCS-EF1-puro* vector (System Biosciences). Based on the DNAJA1-encoding vector, vectors with DNAJA1 mutations of Y7A, K44A, and Q47A were generated using the QuikChange II XL Site-Directed Mutagenesis Kit (Agilent). DNA sequences for generated cDNAs and plasmids were confirmed by sequencing.

Establishment of cells knockout for mutp53 and DNAJA1

DNAJA1- or *p53*-knockout KHOS/NP and CAL33 cells were established, using a *DNAJA1* sgRNA CRISPR lentivector (target #2, target sequence: GAGTGCTGTCCCAATTGCCG; ABM Inc) or a *p53* sgRNA CRISPR lentivector (pXPR003-sgTP53-4, #118022, target sequence: CCCCGGACGATATTGAACAA; Addgene) with an adenoviral vector encoding *eGFP-Cas9* (#1901, VECTOR BIOLABS).

Briefly, cells were simultaneously infected with a sgRNA-encoding lentiviral vector and the *Cas9*-encoding adenoviral vector, followed by G418 (500 µg/ml) or puromycin (1 ng/ml) selection. After single cell colonization, lack of DNAJA1 or p53 protein levels was confirmed by western blotting for each colony. Genomic DNAs were also extracted from the cells and PCR-amplified for the surrounding region of the target site, followed by sequencing to confirm the alternation of coding regions.

Western blotting

Cells were lysed with 1xSDS sample buffer or CelLytic M buffer (C2978, Sigma-Aldrich) containing protease inhibitors (PIA32955, Thermo Fisher). Equal amounts of cell lysate were separated by SDS-PAGE and transferred to polyvinylidene fluoride membrane (GE10600002, MilliporeSigma). Proteins were detected with specific primary antibodies and fluorescent dye-conjugated secondary antibodies. Blots were analyzed with the Azure Sapphire Biomolecular Imager (Azure Biosystems).

Co-immunoprecipitation study

Cells were lysed with IP lysis buffer (PI87788, Pierce) containing protease inhibitors. The whole cell lysate was incubated with anti-HDJ2 antibody or anti-GFP antibody, which were pre-incubated with protein A/G agarose beads (sc-2003, Santa Cruz Biotechnology), overnight at 4˚C on a rotator. The precipitants were subjected to western blotting.

Rac1/Cdc42 pull-down activation assay

To detect active Rac1/Cdc42, the Rac1/Cdc42 pull-down activation assay biochem kit (PAK02-A, Cytoskeleton) was used. Briefly, cell lysates were incubated with the GST-PAK-PBD beads to capture active Cdc42 and Rac1 at 4˚C for 4h. After washing the beads, beads-bound precipitants were incubated with 2xSDS sample buffer at 95˚C for 10 min, followed by western blotting for Rac1 and Cdc42.

Immunofluorescence and F-actin staining

Cells were fixed with 4% paraformaldehyde (15710, Electron Microscopy Sciences) in PBS for 25 min, followed by permeabilization with PBS containing 0.3% Triton X-100 (PBS-T) for 5 min. Permeabilized cells were blocked with 1% bovine serum albumin in PBS-T for 1h and incubated with primary antibody at 4˚C overnight. After washing with PBS, secondary antibodies conjugated with Alexa Fluor dye were added to the cells, followed by incubation at room temperature for 1h.

For F-actin staining, permeabilized cells were incubated with Rhodamine-Phalloidin (00027, Biotum) for 20 min. Cells were mounted with the ProLong™ Gold Antifade Mountant with DAPI (P36935, Thermo Fisher). BZ-X800 fluorescence microscope (KEYENCE) was used to obtain the images. BZ-X800 analyzer (VR-H2AE) was used to count the number of filopodia in the circumference of at least 100 cells. The total number of filopodia was normalized with the circumference, and the percentage of filopodia formation was calculated by dividing the normalized number of filopodia in each group by that in the control.

Transwell migration assay

24-well transwell chambers (6.5mm diameter, 8 μm pore size, 3464, Corning) were used for migration assays. Cells were pretreated with A11 or 0.1% DMSO for 12h on a 6-well plate, followed by counting living cells with trypan blue. Alive cells were suspended with 0.5% FBS-containing DMEM and were seeded in the upper chamber with A11 or 0.1% DMSO, while 10% FBS-containing DMEM was added to the lower chamber as a chemoattractant. Migrating cells were fixed and stained with the Diff-Quik Stain Set (Dade Behring) at 12h after seeding cells (cells were exposed to A11 for 24h in total). Stained cells in the entire field of the membrane were counted. The percentage of migration was calculated by dividing the number of migrating cells in each group by that in the control.

MTT assay

Cells seeded on a 96-well plate were treated with DMSO or A11 (1 nM – 100 μM) for 72h. The cells were then incubated with 3-(4,5-Dimethylthiazol-2-yl)-2,5-Diphenyltetrazolium Bromide (MTT, M6494, Invitrogen) at 37°C for 3h in the 5% CO_2 incubator. The media were replaced with DMSO and incubated for 15 min to dissolve the formazan crystals in the dark. Absorbance at 570 nm and 630 nm in each well was measured using Synergy H4 hybrid plate reader (BioTek® Instruments, Inc., USA).

Cellular thermal shift assay (CETSA)

Cells were treated with DMSO or compounds for 4h. After trypsinization, cells were suspended with PBS and aliquoted into thin-walled PCR tubes (Fisher Scientific, USA). Cells were exposed to the target temperature (37 – 55˚C) for 3 min, followed by 3 cycles

of 3 min freeze and 3 min thaw. After the third thaw, cell lysates were centrifuged at 18,000 g at 4˚C for 1h. The supernatants were subjected to SDS-PAGE and western blotting for the proteins of interest.

Statistical analysis

All statistical analyses were performed using the GraphPad Prism 9 software (GraphPad). The statistical significance for CETSA was assessed by two-way ANOVA. Student's *t*-test was used for migration and filopodia formation assays, as well as Rac1/Cdc42 pull-down activation assays. For all analyses, p-values < 0.05 were considered statistically significant.

Supplementary References

1 Sterling T, Irwin JJ. ZINC 15--Ligand Discovery for Everyone. *J Chem Inf Model* 2015; 55: 2324-2337.

2 Baell JB, Holloway GA. New substructure filters for removal of pan assay interference compounds (PAINS) from screening libraries and for their exclusion in bioassays. *J Med Chem* 2010; 53: 2719-2740.

3 Bruns RF, Watson IA. Rules for identifying potentially reactive or promiscuous compounds. *J Med Chem* 2012; 55: 9763-9772.

4 Hawkins PC, Skillman AG, Warren GL, Ellingson BA, Stahl MT. Conformer generation with OMEGA: algorithm and validation using high quality structures

from the Protein Databank and Cambridge Structural Database. *J Chem Inf Model* 2010; 50: 572-584.

5 Sievers F, Wilm A, Dineen D, Gibson TJ, Karplus K, Li W *et al*. Fast, scalable generation of high-quality protein multiple sequence alignments using Clustal Omega. *Mol Syst Biol* 2011; 7: 539.

6 Stark JL, Mehla K, Chaika N, Acton TB, Xiao R, Singh PK *et al*. Structure and function of human DnaJ homologue subfamily a member 1 (DNAJA1) and its relationship to pancreatic cancer. *Biochemistry* 2014; 53: 1360-1372.

7 Johnson DK, Karanicolas J. Selectivity by small-molecule inhibitors of protein interactions can be driven by protein surface fluctuations. *PLoS Comput Biol* 2015; 11: e1004081.

8 Johnson DK, Karanicolas J. Ultra-High-Throughput Structure-Based Virtual Screening for Small-Molecule Inhibitors of Protein-Protein Interactions. *J Chem Inf Model* 2016; 56: 399-411.

9 Hawkins PC, Skillman AG, Nicholls A. Comparison of shape-matching and docking as virtual screening tools. *J Med Chem* 2007; 50: 74-82.

10 Leaver-Fay A, Tyka M, Lewis SM, Lange OF, Thompson J, Jacak R *et al*. ROSETTA3: an object-oriented software suite for the simulation and design of macromolecules. *Methods Enzymol* 2011; 487: 545-574.

Supplementary Figure legends

Supplementary Fig. 1 7-3 decreases protein levels of conformational mutp53 and DNAJA1 in a manner dependent on the concentration and treatment period. **A-D** Representative western blotting (upper) and immunofluorescence (lower) for p53, DNAJA1, and GAPDH or DAPI using HN31 (A, B) or MG63-p53^{R175H} cells (C, D) treated with DMSO (D) or 7-3 at different concentrations for 48h (A, C) and at 40 μM for different treatment periods (B, D). Scale bar: 50 μm.

Supplementary Fig. 2 A11 decreases protein levels of conformational mutp53 and DNAJA1 in a concentration- and treatment time-dependent manner. **A** Representative western blotting (upper) and immunofluorescence (lower) for p53, DNAJA1, and GAPDH using FaDu (p53^{R248L}), HSC4 (p53^{R248Q}), SJSA1 (p53^{wt}), U2OS (p53^{wt}), MG63 (p53^{null}), and HCT116 (p53$^{-/-}$) cells treated with DMSO (D) or A11 at 20 μM for 24h. Scale bar: 50 μm. **B-E** Representative western blotting for p53, DNAJA1, and GAPDH using CAL33 (B, C) and MG63-p53^{R175H} (D, E) cells treated with DMSO (D) or A11 at different concentrations for 24h (B, D) and at 20 μM for different treatment periods (C, E).

Supplementary Fig. 3 A11 decreases protein levels of DNAJA1 and subsequently conformational mutp53 without altering the binding between DNAJA1 and conformational mutp53 as well as mRNA levels of DNAJA1 and mutp53. **A** Representative western blotting for DNAJA1, p53, and GAPDH (left) and a summarized graph showing normalized DNAJA1 and p53 band densities to those in GAPDH, using KHOS/NP cells treated with A11 at 20 μM for different treatment periods (right). Mean ±

SEM (n=3). **B** Representative western blotting for DNAJA1, p53, and GAPDH using control and DNAJA1-KO CAL33 cells treated with DMSO (D) or A11 at 20 μM for 24h. **C** Representative results of co-immunoprecipitation studies with a DNAJA1 antibody using CAL33 cells treated with DMSO (D) or A11 (A) at 20 μM for 12h. A GFP antibody was used as an isotype control. **D**, **E** qPCR analysis for *DNAJA1* and *p53* using KHOS/NP (D) or Huh7 (E) cells treated with DMSO or A11 at different concentrations for 24h. Mean ± SEM (n=3). n.s.: not significant; two-tailed Student's *t*-test. **F**, **G** qPCR analysis for p53 downstream target genes including *CDKN1A, BBC3*, and *MDM2* using KHOS/NP (F) or Huh7 (G) cells treated with DMSO or A11 at different concentrations for 24h. Mean ± SEM (n=3). n.s.: not significant; two-tailed Student's *t*-test.

Supplementary Fig. 4 A11 has minimal effect on migratory potential in cancer cells with DNA contact mutp53, wtp53, and p53 null. **A** Summary of 72h-IC_{50} values of A11 in control, DNAJA1-KO, and p53-KO CAL33 cells, determined by MTT assays. Mean ± SEM (n=5). **B** Representative image of transwell migration assays (upper) and summarized graphs (lower), using cell lines with DNA-contact mutp53 (MDA-MB-231, FaDu, HSC4), wtp53 (SJSA1, U2OS, HCT116 $p53^{+/+}$), and p53 null (MG63, HCT116 $p53^{-/-}$, H1299), treated with A11 at 20 μM for 12h. Cells were pre-treated with A11 for 12h. Mean ± SEM (n=3). n.s.: not significant; two-tailed Student's *t*-test. Scale bar: 100 μm. **C** Representative images of F-actin staining (upper) and a summarized graph of relative filopodia-forming potential (lower) using cell lines with DNA contact mutp53 (MDA-MB-231, FaDu, HSC4), wtp53 (SJSA1, U2OS, HCT116 $p53^{+/+}$), p53 null (MG63,

HCT116 p53$^{-/-}$, H1299) cells, treated with A11 at 20 µM for 24h. Mean ± SEM (n=3). n.s.: not significant; two-tailed Student's *t*-test. Scale bar: 50 µm.

Supplementary Fig. 5 A11 decreases protein levels of other HSP40/JDP members. **A** Representative western blotting for several HSP40/JDP members and loading controls (GAPDH, alpha-tubulin, Vinculin) using CAL33 cells treated with DMSO (D) or A11 (A) at 20 µM for 24h. **B** Representative western blotting for several HSP40/JDP members (A11 responders: DNAJA1, DNAJB6; A11 non-responders: DNAJC6, DNAJC10) and loading controls (GAPDH, Vinculin) using CAL33-control, DNAJA1-KO, and p53-KO cells treated with DMSO (D) or A11 (A) at 20 µM for 24h.

Supplementary Fig. 6 A triple mutant DNAJA1 (Y7A/K44A/Q47A: mutDNAJA1) does not respond to A11. **A** Representative western blotting for exogenous DNAJA1, as well as endogenous DNAJA1 (control lanes only), DNAJB6, DNAJC6, p53 (R156P), and GAPDH, using control and DNAJA1-KO (JA1-KO) KHOS/NP cells with or without exogenous expression of wtDNAJA1 or mutDNAJA1, treated with DMSO (D) or A11 (A) at 20 µM for 24h. **B** Representative images of immunofluorescence staining for p53, DNAJA1, and DAPI using the same experimental set of KHOS/NP sub-cell lines as in Fig. S6A. Scale bar: 50 µm.
