# Supplementary material for: DNAJA1- and conformational mutant p53-dependent inhibition of cancer cell migration by a novel compound identified through a virtual screen
Source: Cell Death Discov. 2022 Oct 31;8:437. doi: 10.1038/s41420-022-01229-5 (PMC9622836; doi:10.1038/s41420-022-01229-5)

Supplementary Figure S1

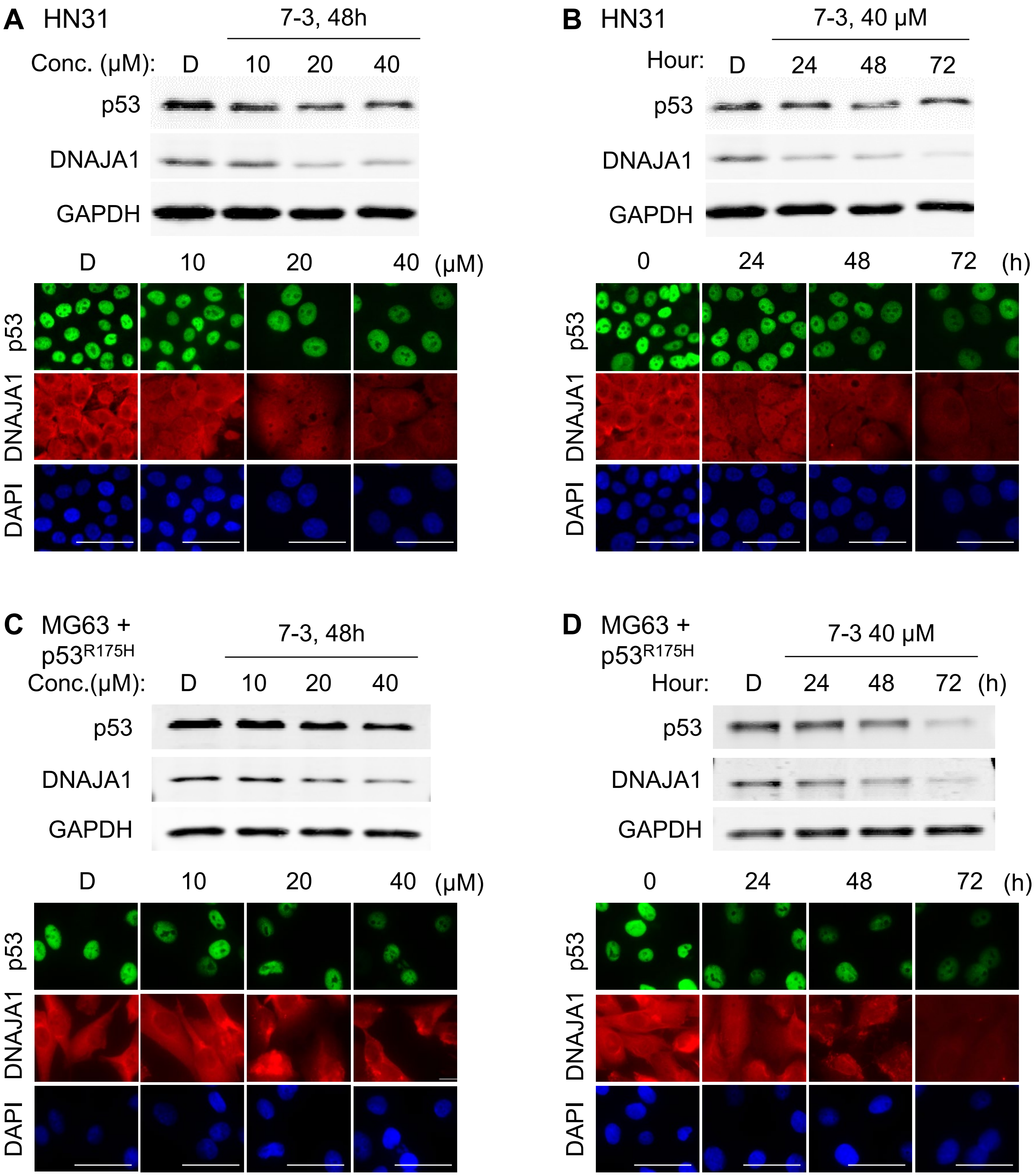

Supplementary Figure S2

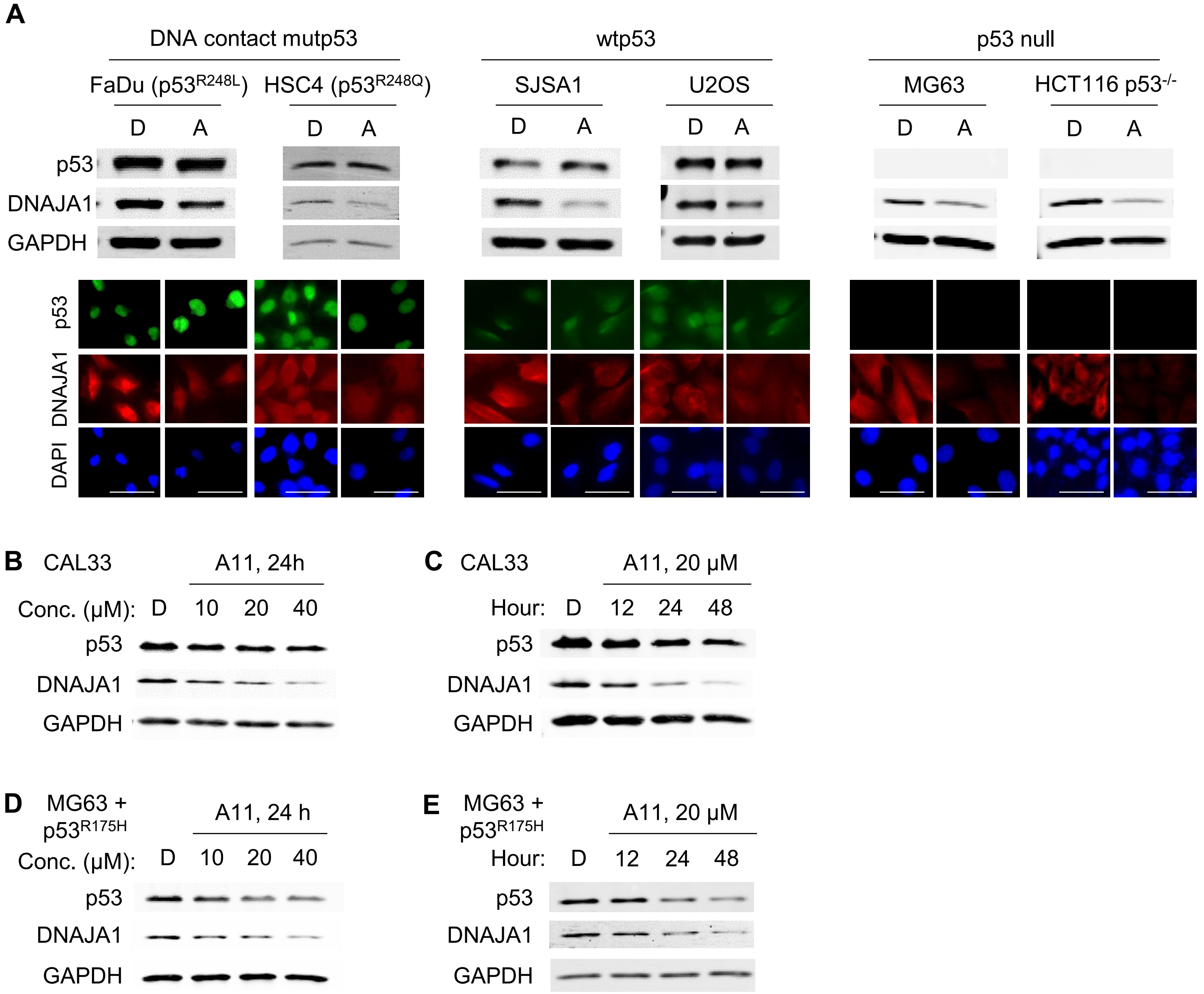

Supplementary Figure S3

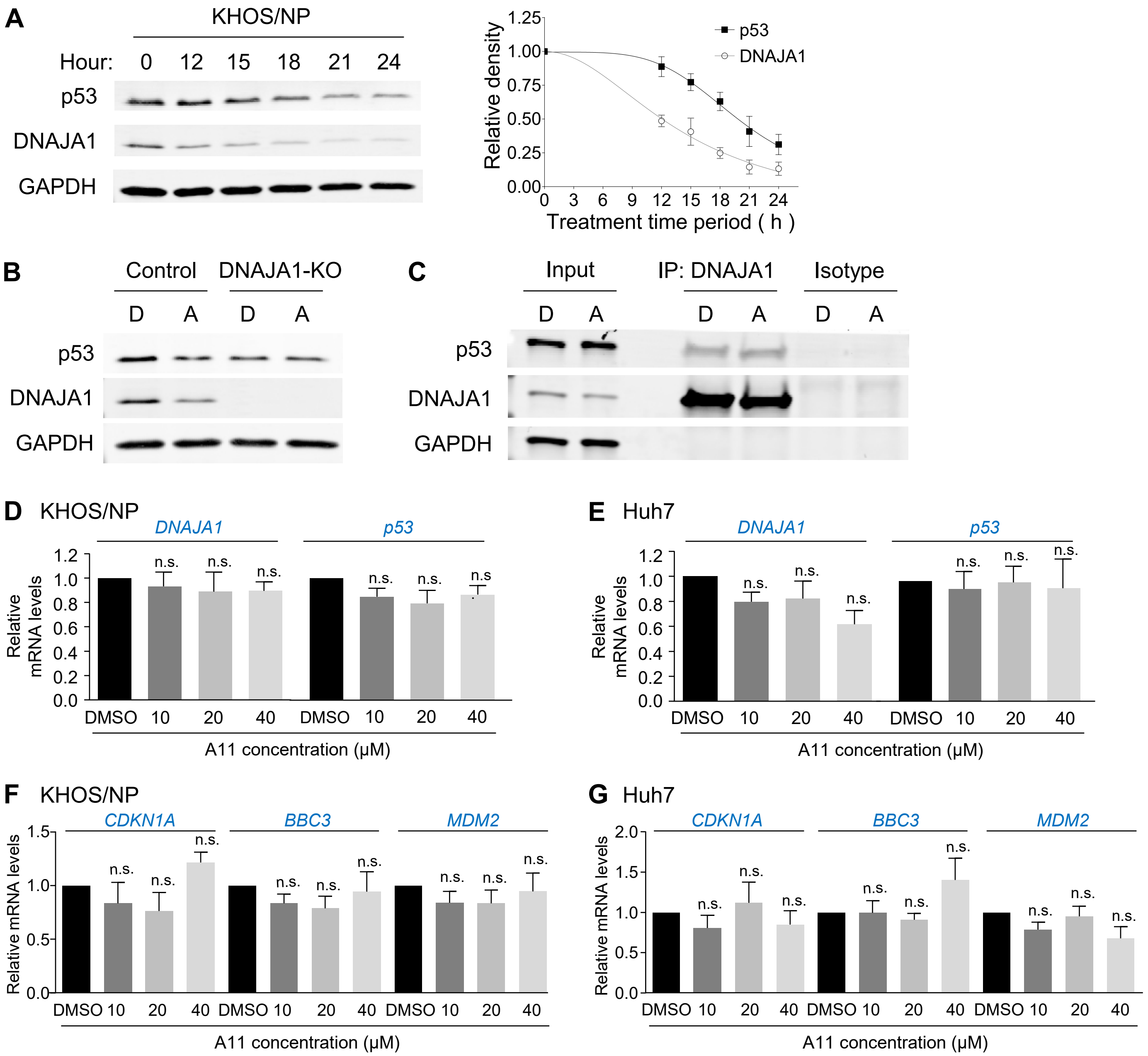

Supplementary Figure S4

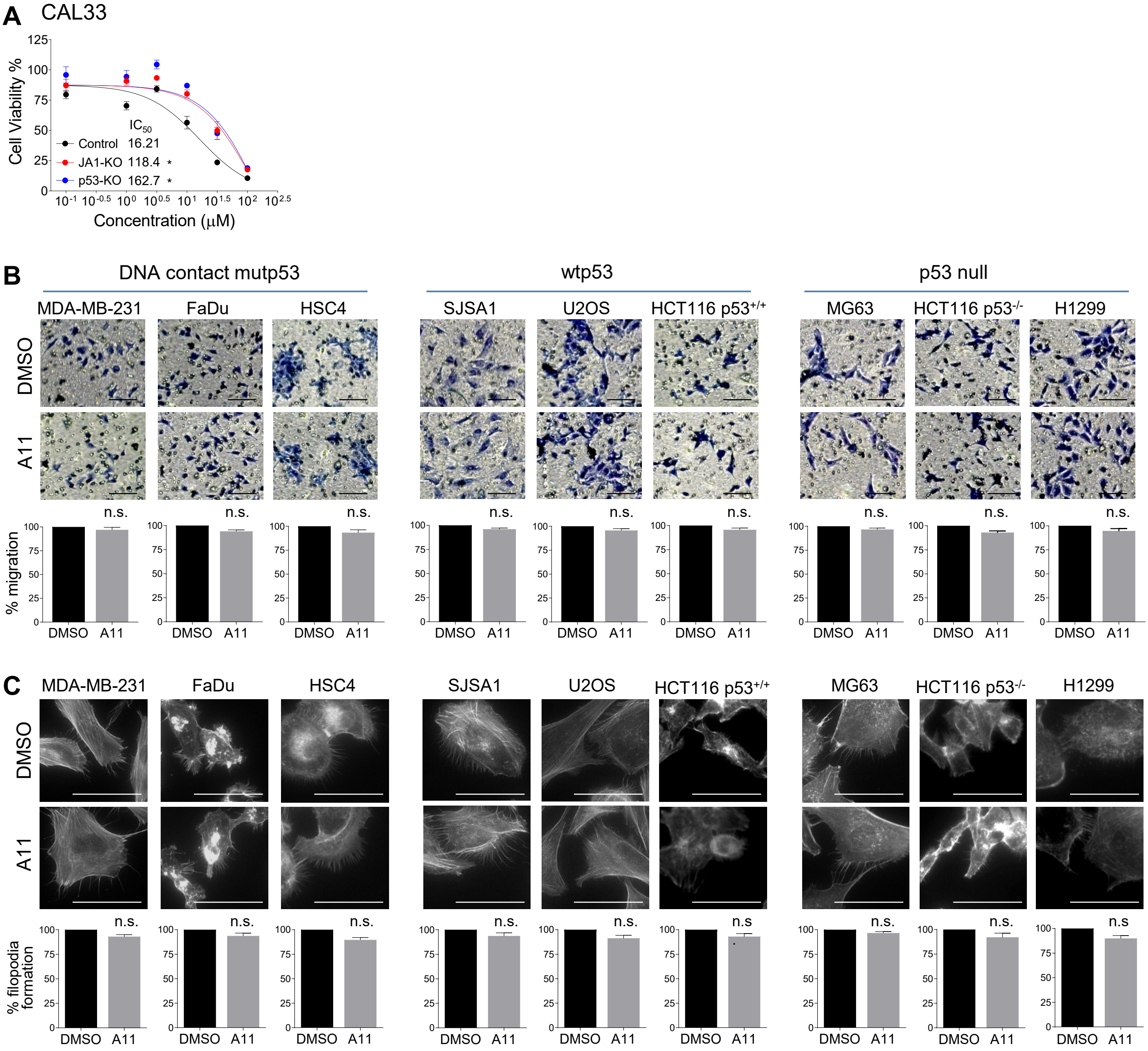

Supplementary Figure S5

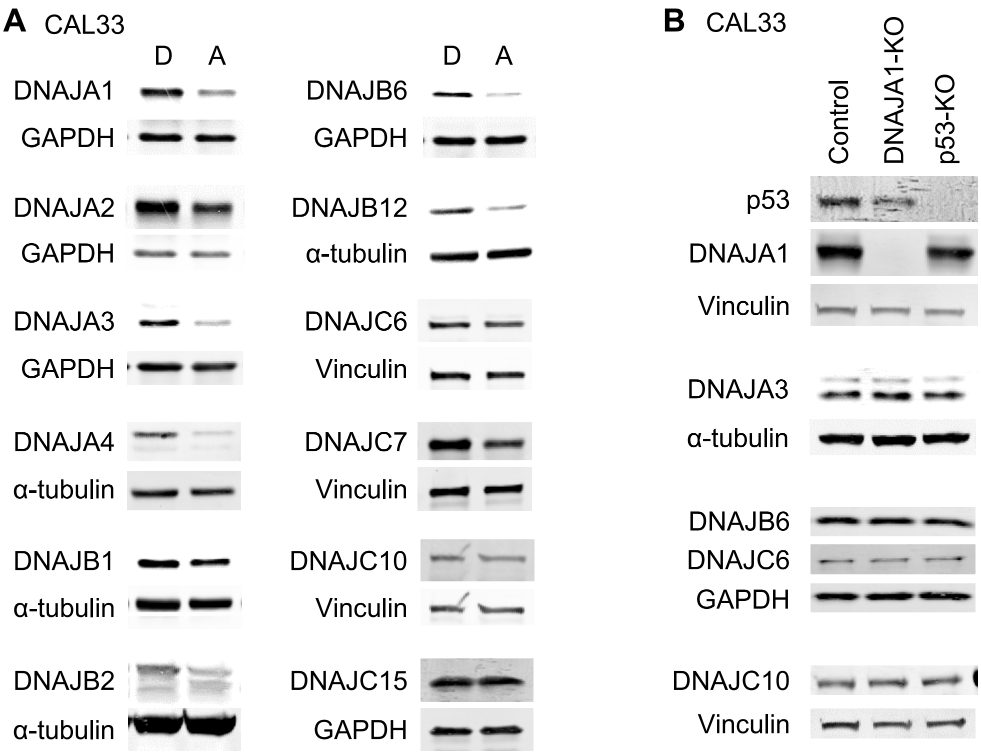

Supplementary Figure S6

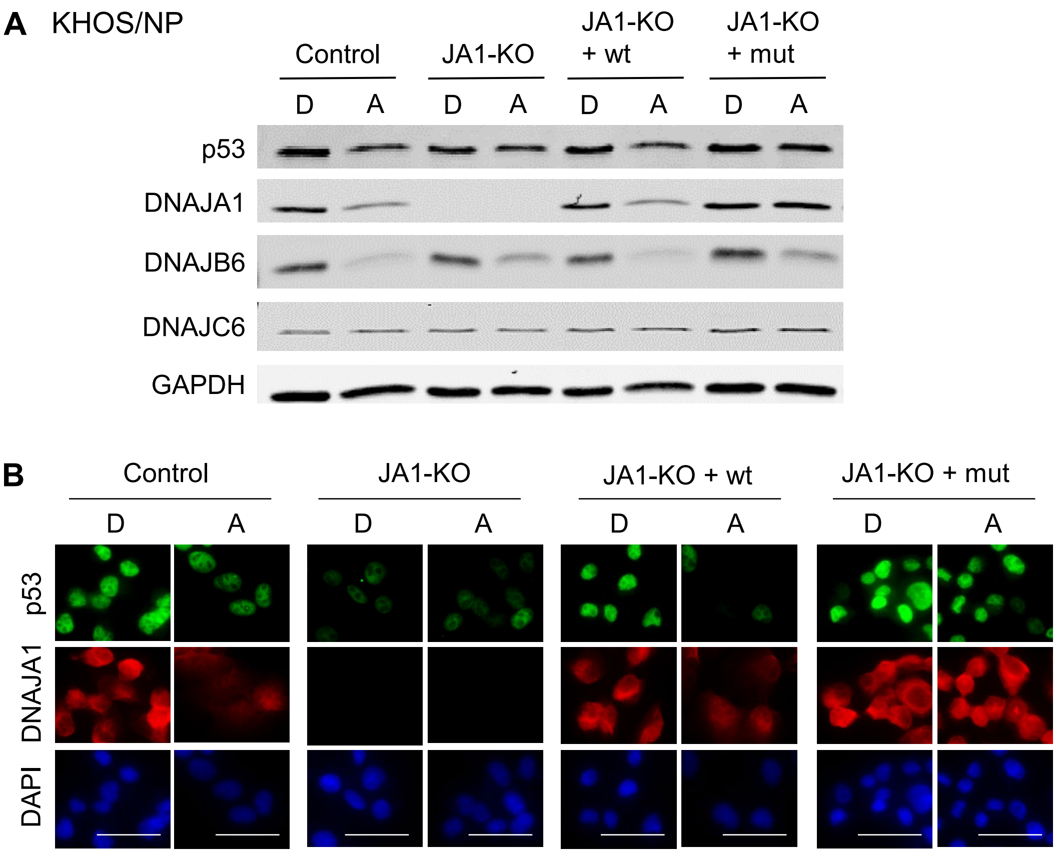

Supplement: Supplementary file 2 — Supplementary Figures [file 41420_2022_1229_MOESM2_ESM.pdf]
