# Supplementary material for: DNAJA1- and conformational mutant p53-dependent inhibition of cancer cell migration by a novel compound identified through a virtual screen
Source: Cell Death Discov. 2022 Oct 31;8:437. doi: 10.1038/s41420-022-01229-5 (PMC9622836; doi:10.1038/s41420-022-01229-5)

Fig. 1C

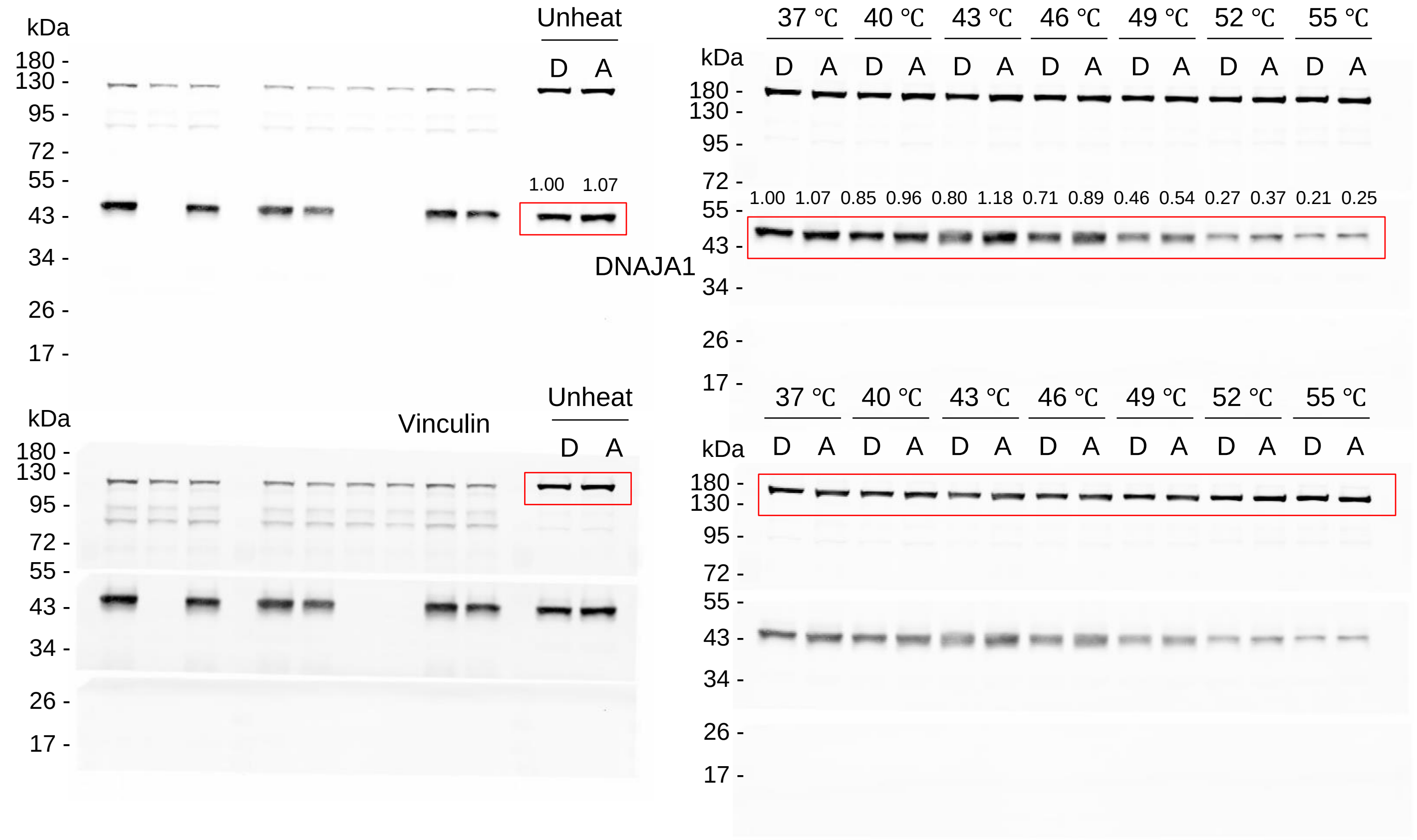

Unheat
D A
1.00 1.07
DNAJA1
Vinculin
37 ℃ 40 ℃ 43 ℃ 46 ℃ 49 ℃ 52 ℃ 55 ℃
D A D A D A D A D A D A D A
1.00 1.07 0.85 0.96 0.80 1.18 0.71 0.89 0.46 0.54 0.27 0.37 0.21 0.25
kDa
180 - 130 - 95 - 72 - 55 - 43 - 34 - 26 - 17 -

Fig. 1D

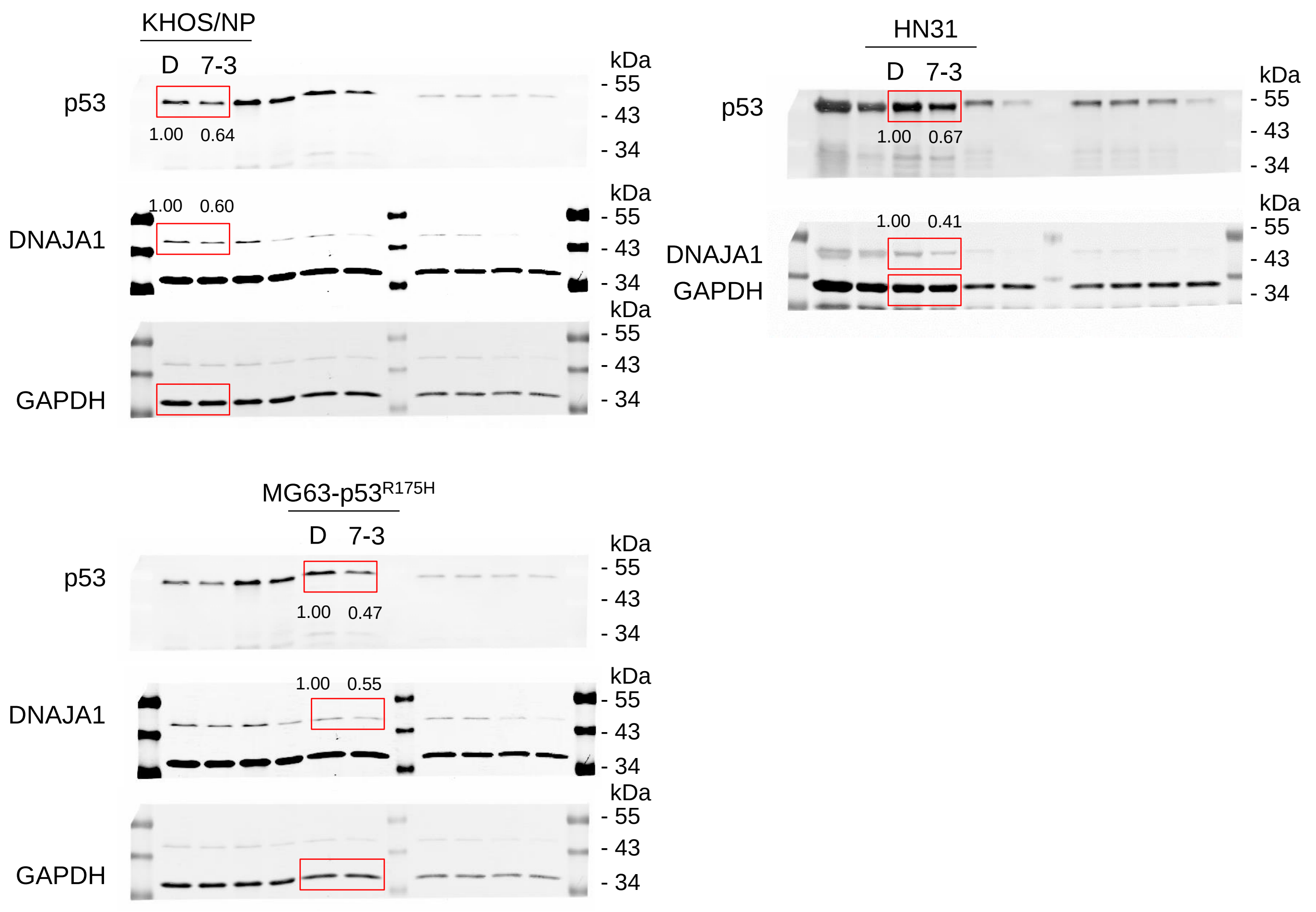

KHOS/NP
D 7-3
p53
1.00 0.64
DNAJA1
1.00 0.60
GAPDH
HN31
D 7-3
p53
1.00 0.67
DNAJA1
1.00 0.41
GAPDH
MG63-p53R175H
D 7-3
p53
1.00 0.47
DNAJA1
1.00 0.55
GAPDH
kDa
- 55 - 43 - 34

Fig. 1E

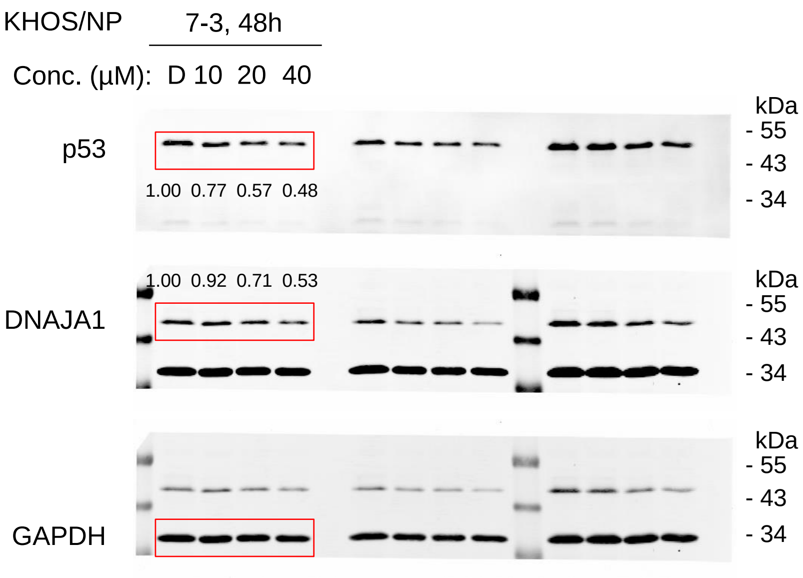
KHOS/NP
7-3, 48h
Conc. (µM): D 10 20 40
p53
1.00 0.77 0.57 0.48
DNAJA1
1.00 0.92 0.71 0.53
GAPDH
kDa
- 55
- 43
- 34

Fig. 1F

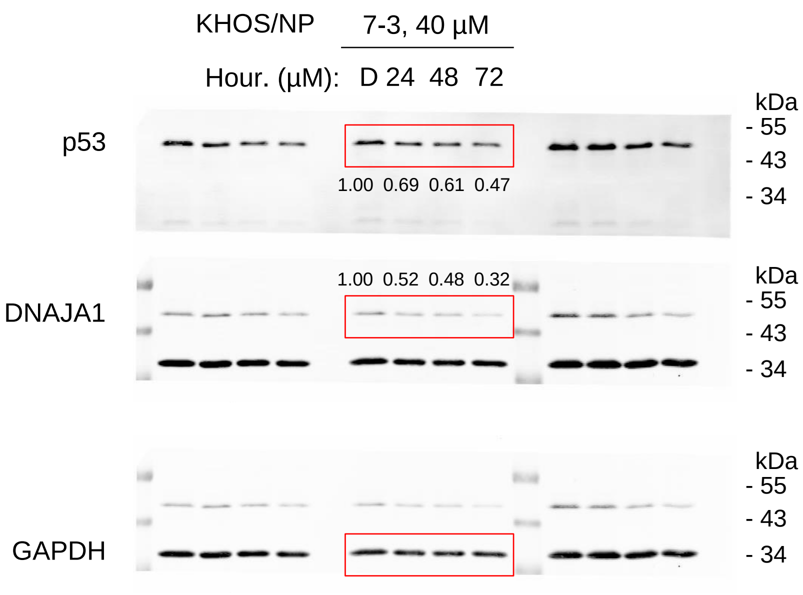
KHOS/NP
7-3, 40 µM
Hour. (µM): D 24 48 72
p53
1.00 0.69 0.61 0.47
DNAJA1
1.00 0.52 0.48 0.32
GAPDH
kDa
- 55
- 43
- 34

Fig. 2B

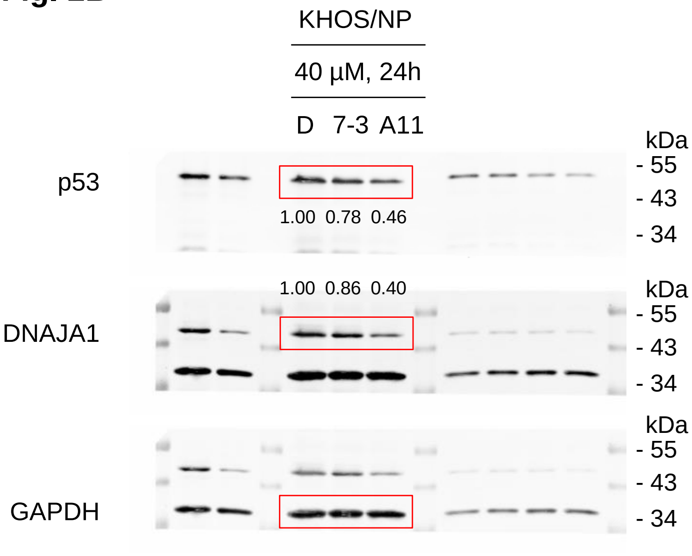
KHOS/NP
40 µM, 24h
D 7-3 A11
p53
1.00 0.78 0.46
DNAJA1
1.00 0.86 0.40
GAPDH
kDa
- 55
- 43
- 34

Fig. 2C

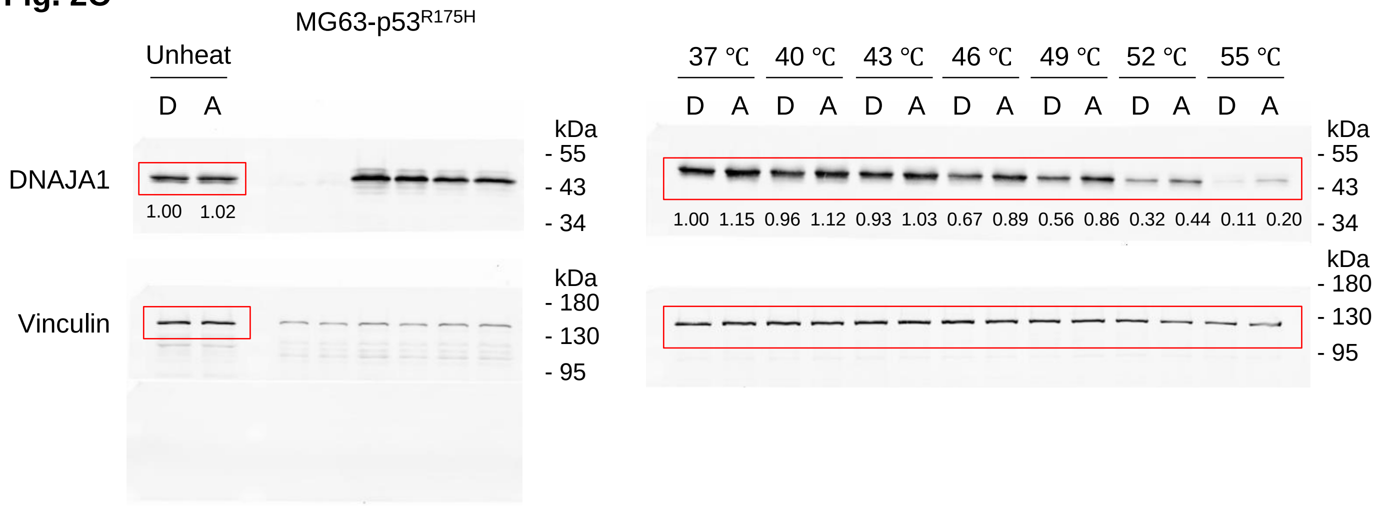
MG63-p53R175H
Unheat
D A
DNAJA1
1.00 1.02
Vinculin
37 ℃ 40 ℃ 43 ℃ 46 ℃ 49 ℃ 52 ℃ 55 ℃
D A D A D A D A D A D A D A
1.00 1.15 0.96 1.12 0.93 1.03 0.67 0.89 0.56 0.86 0.32 0.44 0.11 0.20
kDa
- 55
- 43
- 34
kDa
- 180
- 130
- 95

Fig. 2D

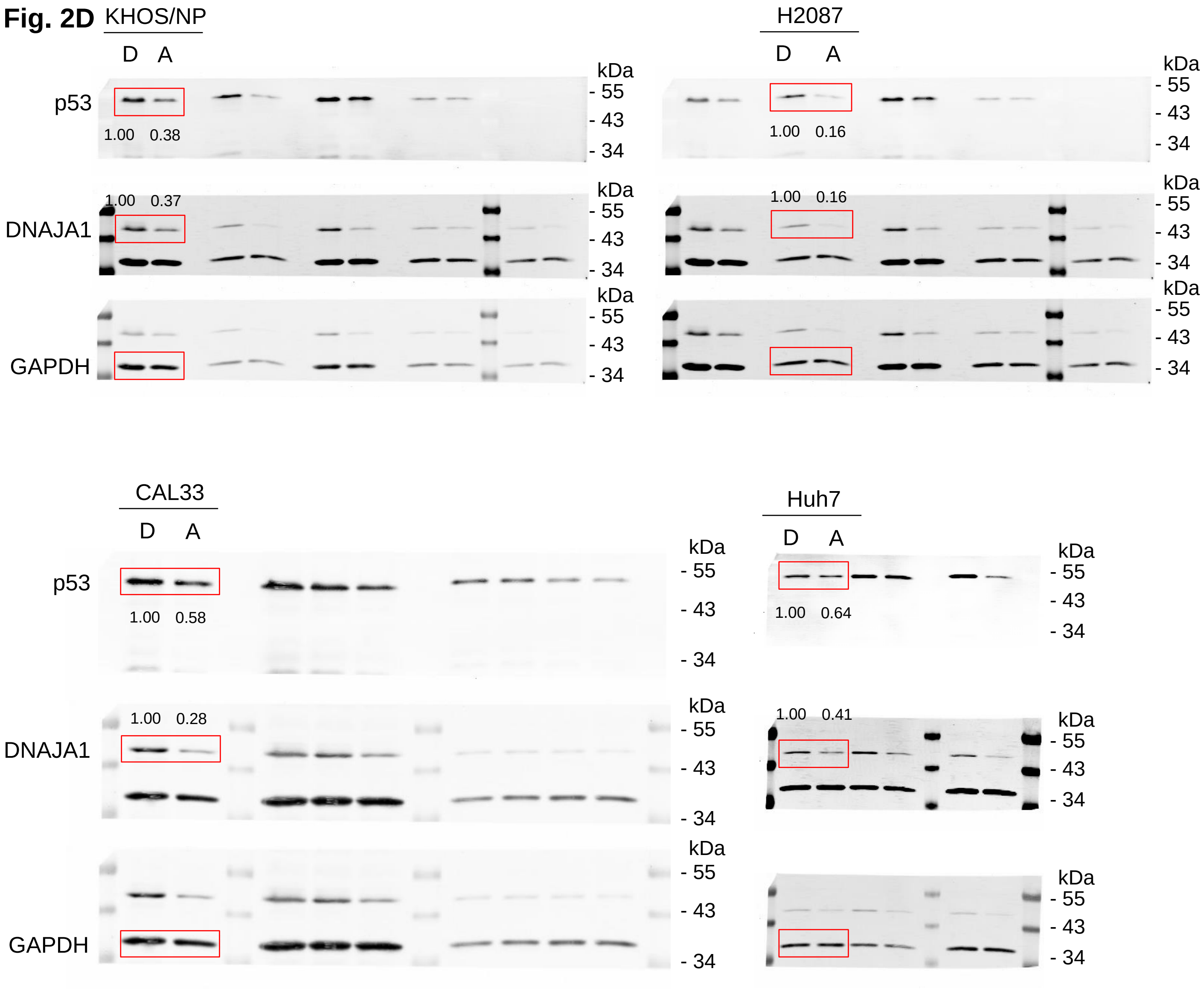

Fig 2E

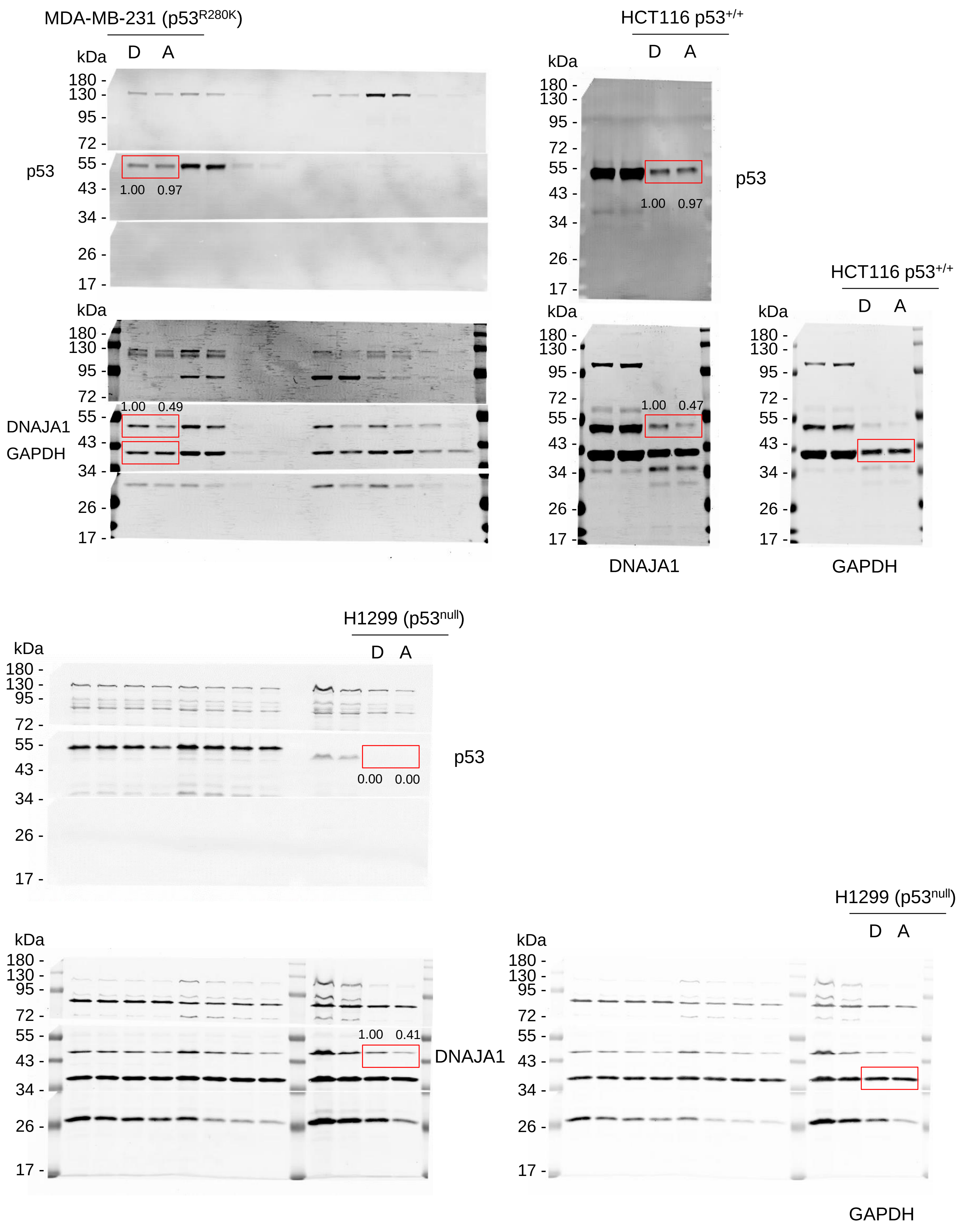
MDA-MB-231 (p53R280K)
kDa
D
A
180 -
130 -
95 -
72 -
55 -
43 -
34 -
26 -
17 -
p53
1.00
0.97
DNAJA1
GAPDH
1.00
0.49
HCT116 p53+/+
p53
1.00
0.97
1.00
0.47
DNAJA1
GAPDH
H1299 (p53null)
p53
0.00
0.00
1.00
0.41
DNAJA1
GAPDH

Fig 2F

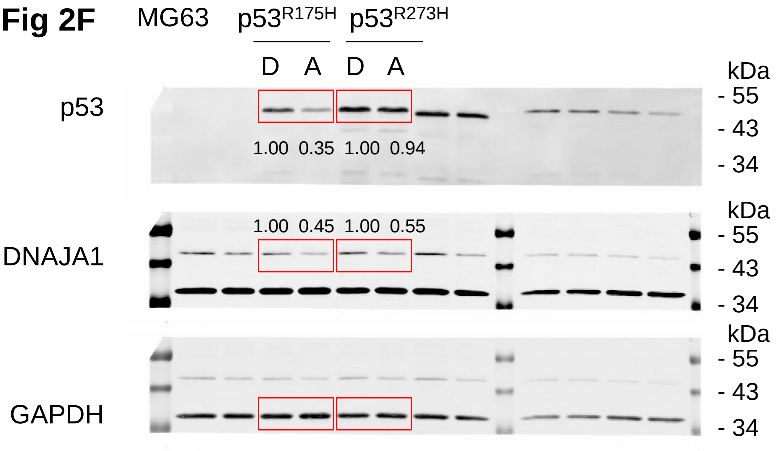

Fig 2G

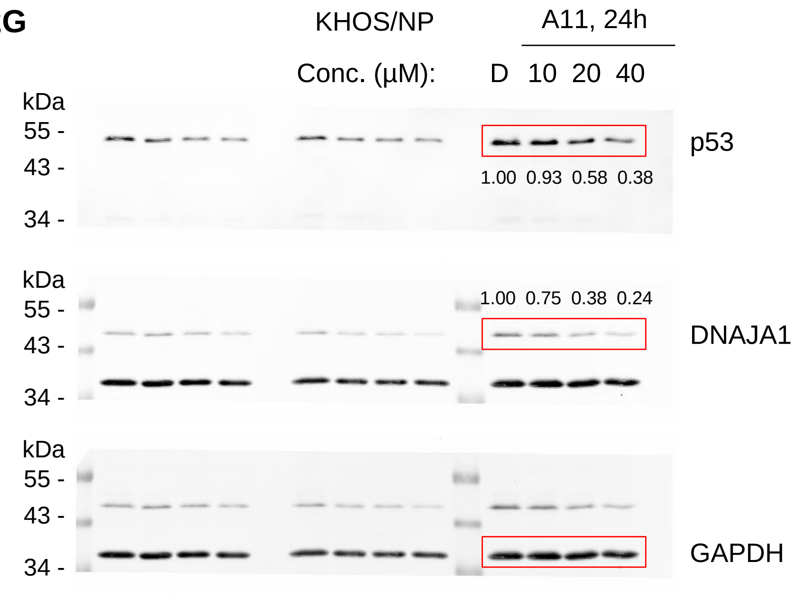

Fig 2H

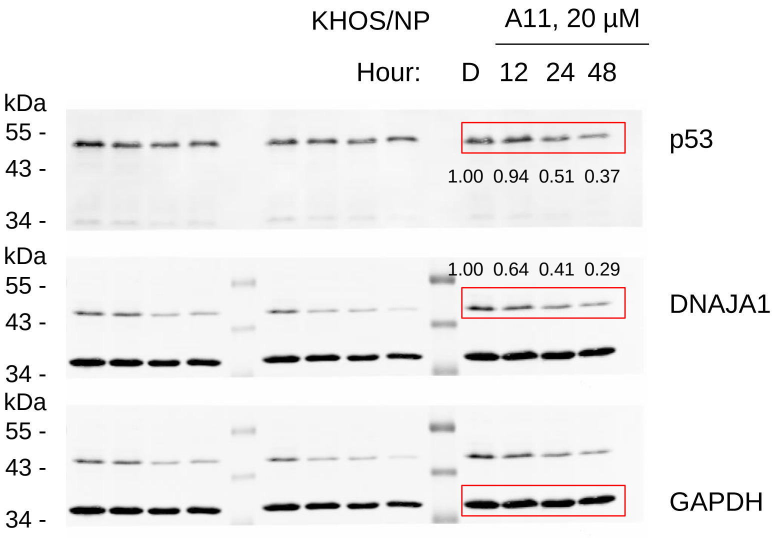

Fig 3A

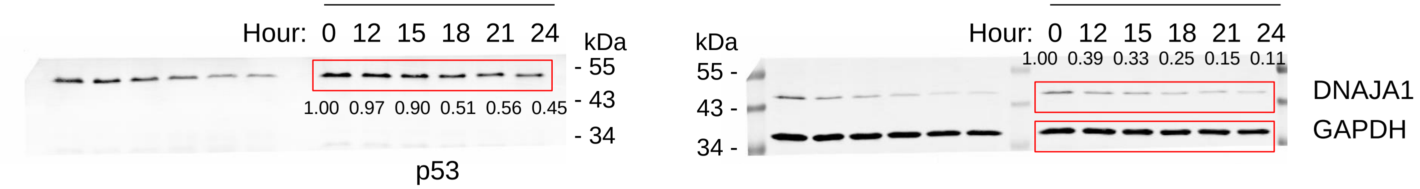

Fig 3B

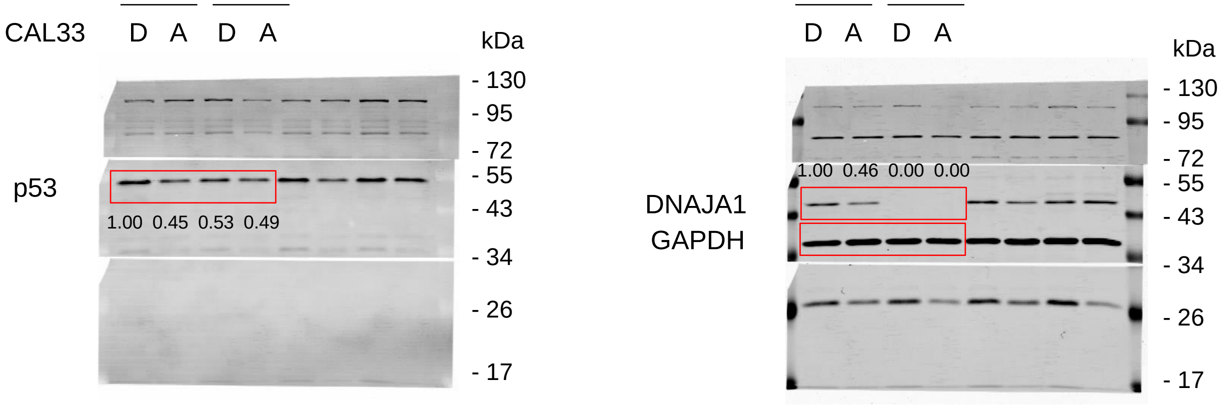

Fig 3C

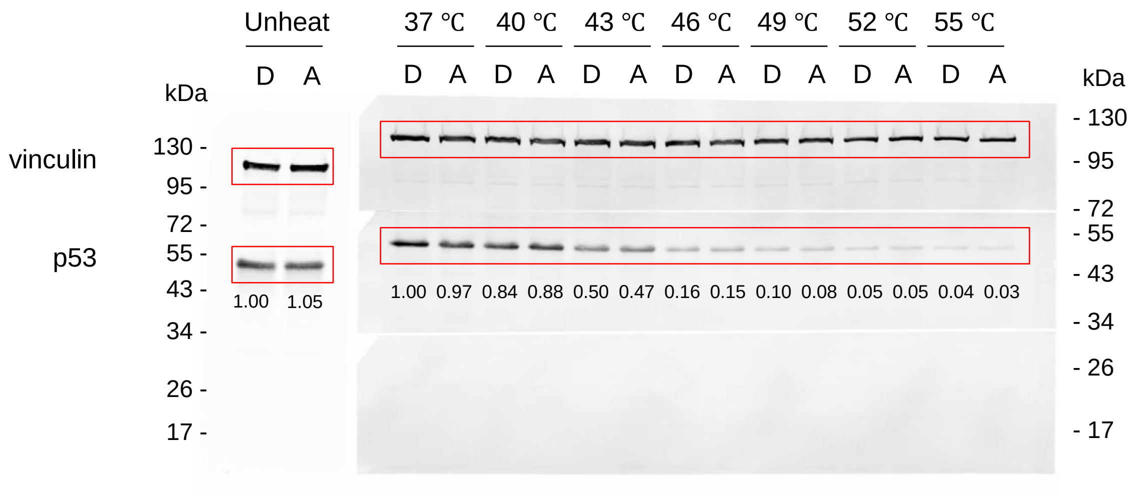

Fig 3D

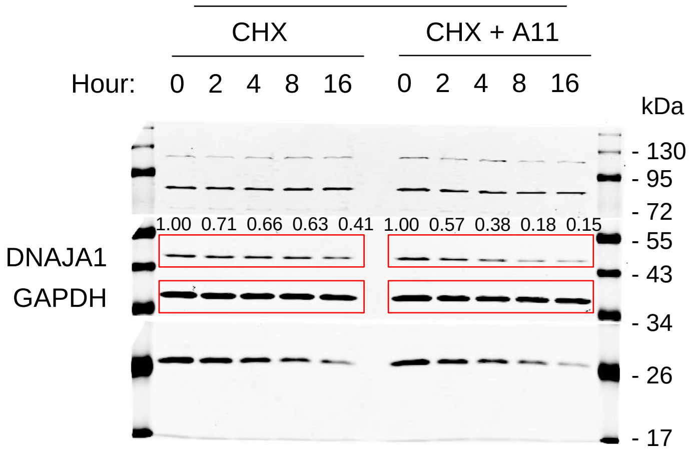

Fig 3E

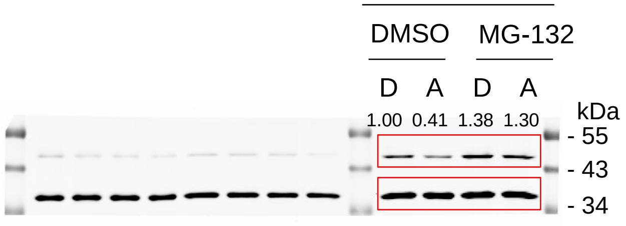

Upper, DNAJA1

Lower, GAPDH

Fig 4E

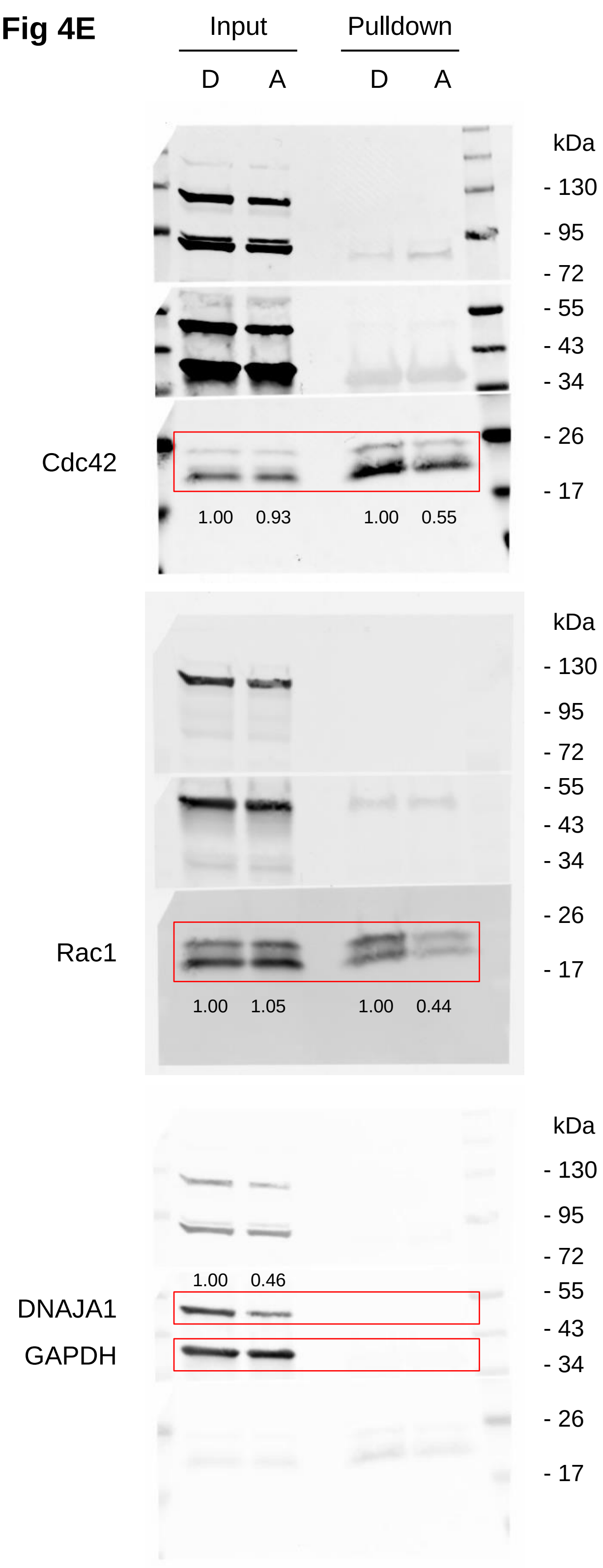
Input
Pulldown
D
A
D
A
kDa
- 130
- 95
- 72
- 55
- 43
- 34
- 26
- 17
Cdc42
1.00
0.93
1.00
0.55
kDa
- 130
- 95
- 72
- 55
- 43
- 34
- 26
- 17
Rac1
1.00
1.05
1.00
0.44
kDa
- 130
- 95
- 72
- 55
- 43
- 34
- 26
- 17
1.00
0.46
DNAJA1
GAPDH

Fig 5A

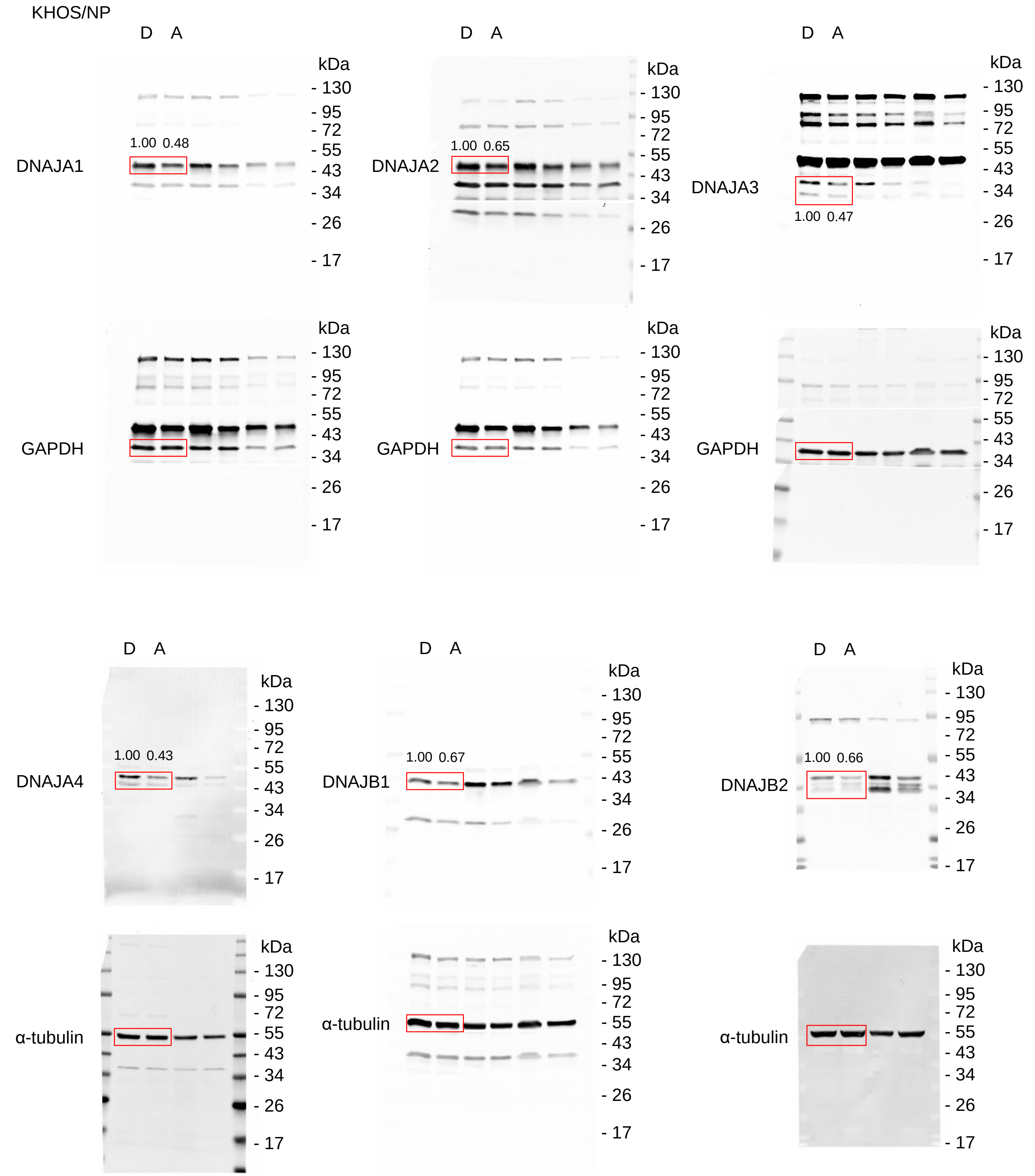

KHOS/NP
D A
DNAJA1
1.00 0.48
DNAJA2
1.00 0.65
DNAJA3
1.00 0.47
GAPDH
DNAJA4
1.00 0.43
DNAJB1
1.00 0.67
DNAJB2
1.00 0.66
α-tubulin
kDa
- 130
- 95
- 72
- 55
- 43
- 34
- 26
- 17

Fig 5A

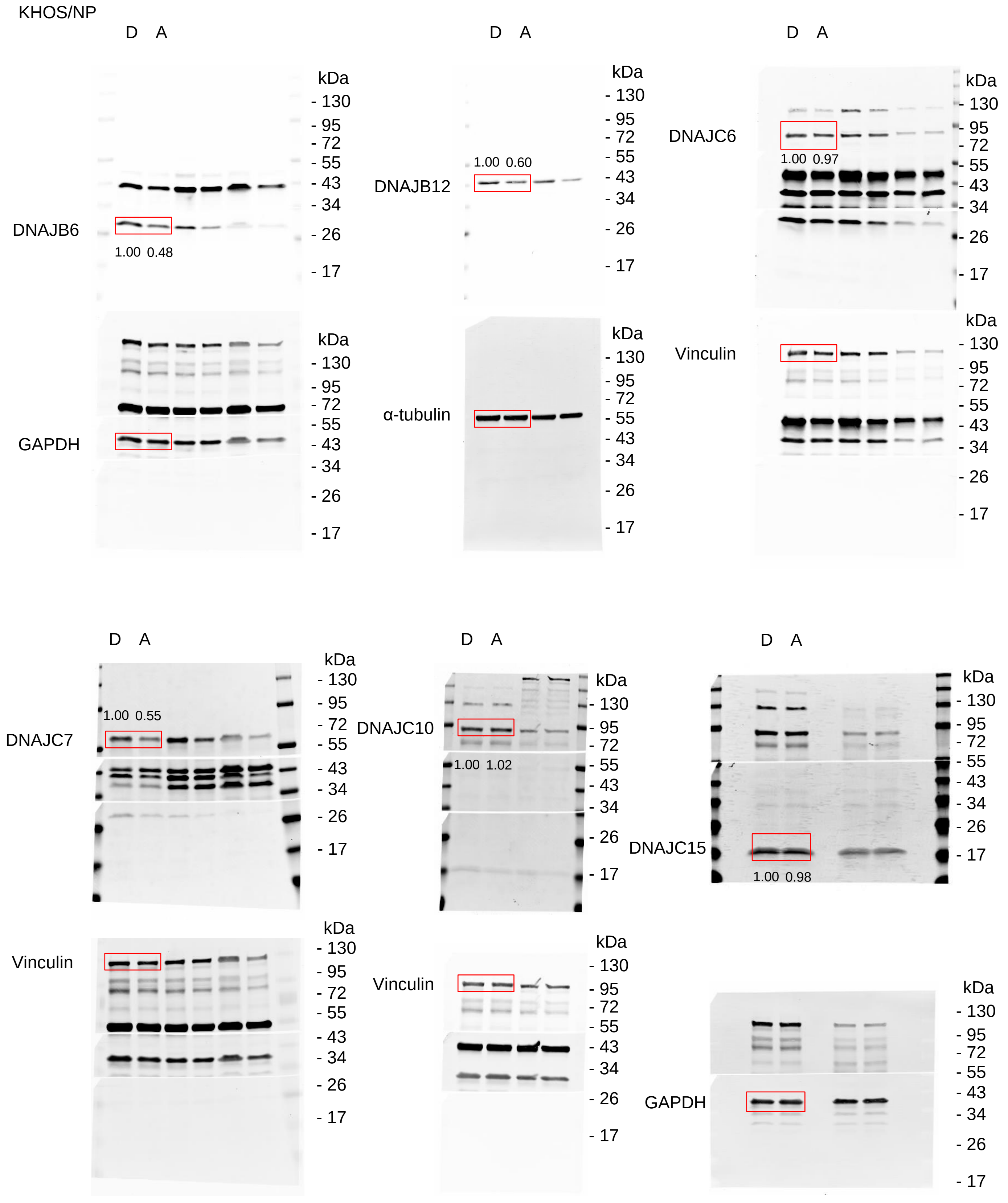
KHOS/NP
D A
DNAJB6
1.00 0.48
GAPDH
DNAJB12
1.00 0.60
α-tubulin
DNAJC6
1.00 0.97
Vinculin
DNAJC7
1.00 0.55
Vinculin
DNAJC10
1.00 1.02
Vinculin
DNAJC15
1.00 0.98
GAPDH
kDa
- 130
- 95
- 72
- 55
- 43
- 34
- 26
- 17

Fig 5C

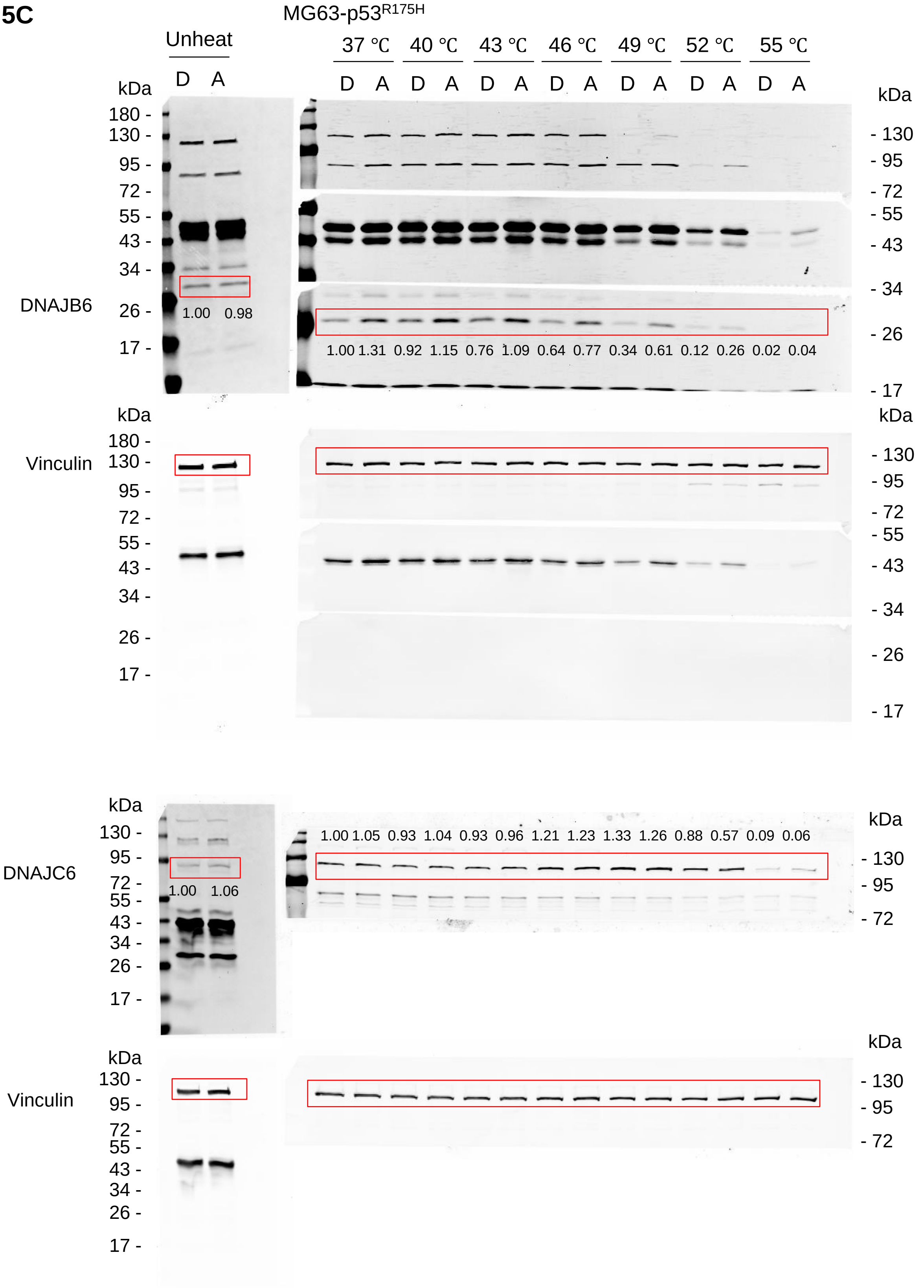
MG63-p53R175H
Unheat
D A
37 ℃
40 ℃
43 ℃
46 ℃
49 ℃
52 ℃
55 ℃
D A D A D A D A D A D A D A
kDa
180 -
130 -
95 -
72 -
55 -
43 -
34 -
26 -
17 -
DNAJB6
1.00 0.98
1.00 1.31 0.92 1.15 0.76 1.09 0.64 0.77 0.34 0.61 0.12 0.26 0.02 0.04
- 130
- 95
- 72
- 55
- 43
- 34
- 26
- 17
Vinculin
DNAJC6
1.00 1.06
1.00 1.05 0.93 1.04 0.93 0.96 1.21 1.23 1.33 1.26 0.88 0.57 0.09 0.06

Fig 6A

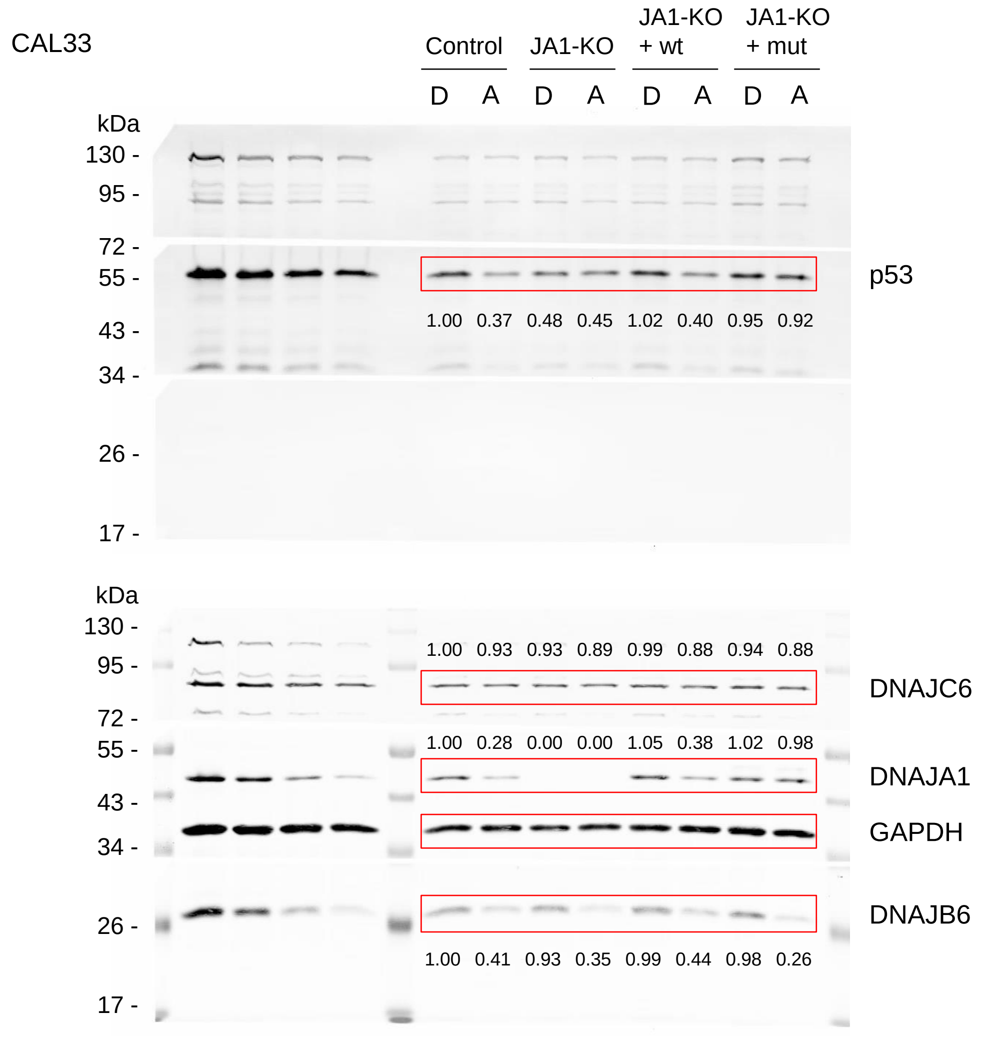
CAL33
Control
JA1-KO
JA1-KO + wt
JA1-KO + mut
D A D A D A D A
kDa
130 -
95 -
72 -
55 -
43 -
34 -
26 -
17 -
p53
1.00 0.37 0.48 0.45 1.02 0.40 0.95 0.92
kDa
130 -
95 -
72 -
55 -
43 -
34 -
26 -
17 -
1.00 0.93 0.93 0.89 0.99 0.88 0.94 0.88
DNAJC6
1.00 0.28 0.00 0.00 1.05 0.38 1.02 0.98
DNAJA1
GAPDH
DNAJB6
1.00 0.41 0.93 0.35 0.99 0.44 0.98 0.26

Fig 6E

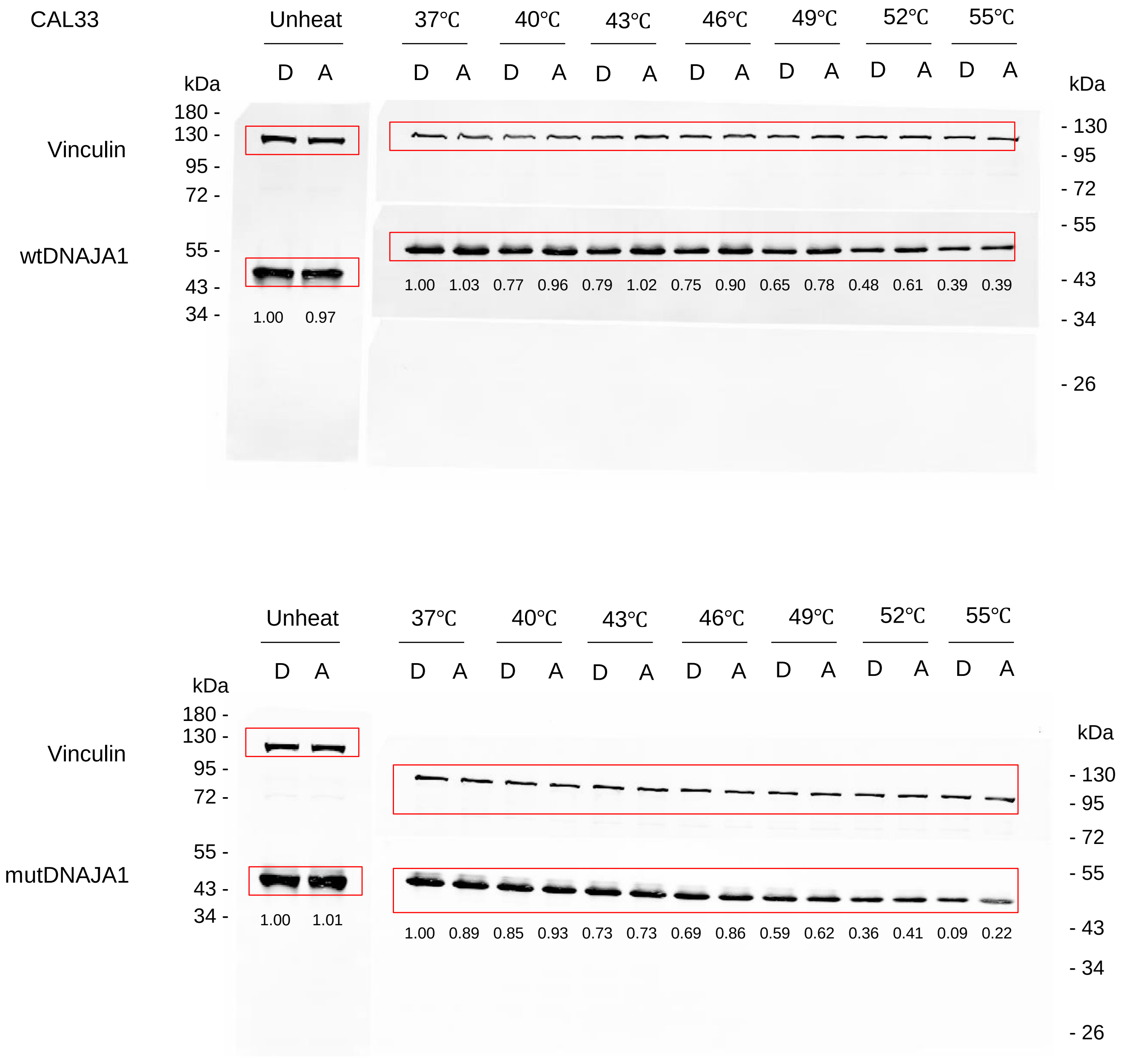
CAL33
Unheat
37℃
40℃
43℃
46℃
49℃
52℃
55℃
D A D A D A D A D A D A D A D A
kDa
180 -
130 -
95 -
72 -
55 -
43 -
34 -
Vinculin
wtDNAJA1
1.00 0.97
1.00 1.03 0.77 0.96 0.79 1.02 0.75 0.90 0.65 0.78 0.48 0.61 0.39 0.39
kDa
- 130
- 95
- 72
- 55
- 43
- 34
- 26
Unheat
37℃
40℃
43℃
46℃
49℃
52℃
55℃
D A D A D A D A D A D A D A D A
kDa
180 -
130 -
95 -
72 -
55 -
43 -
34 -
Vinculin
mutDNAJA1
1.00 1.01
1.00 0.89 0.85 0.93 0.73 0.73 0.69 0.86 0.59 0.62 0.36 0.41 0.09 0.22
kDa
- 130
- 95
- 72
- 55
- 43
- 34
- 26

Supplementary Figure S1

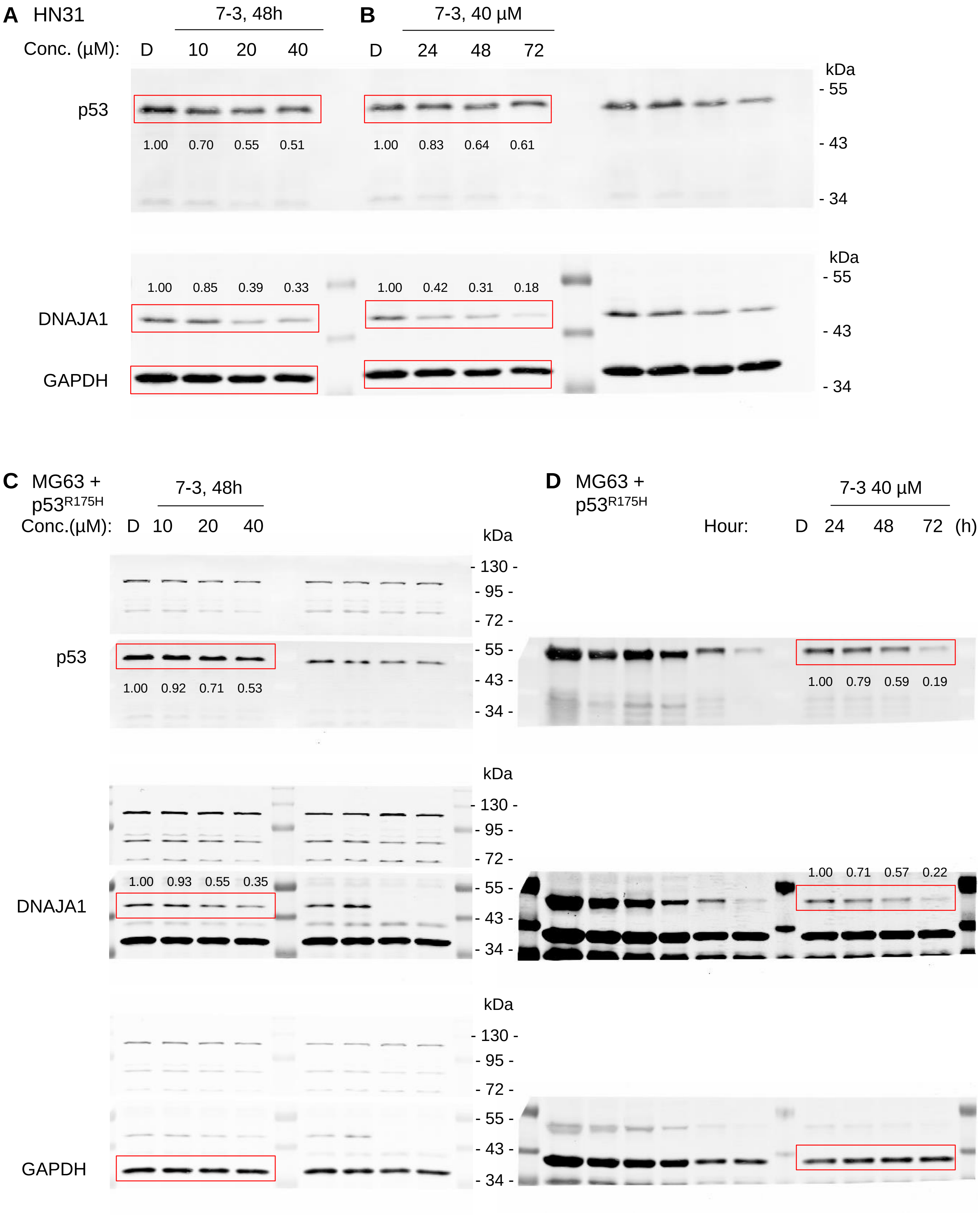

Supplementary Fig S2

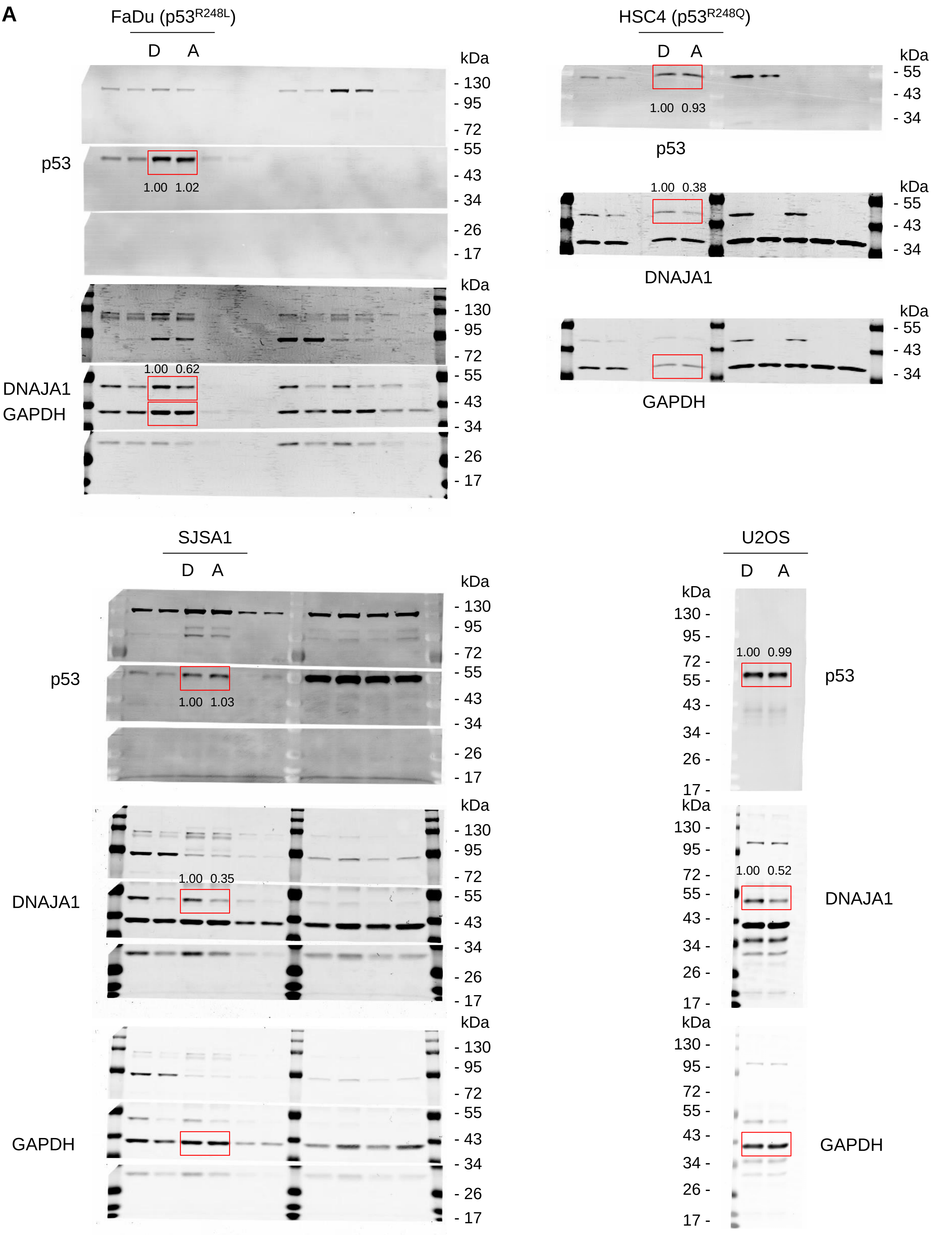

Supplementary Fig S2

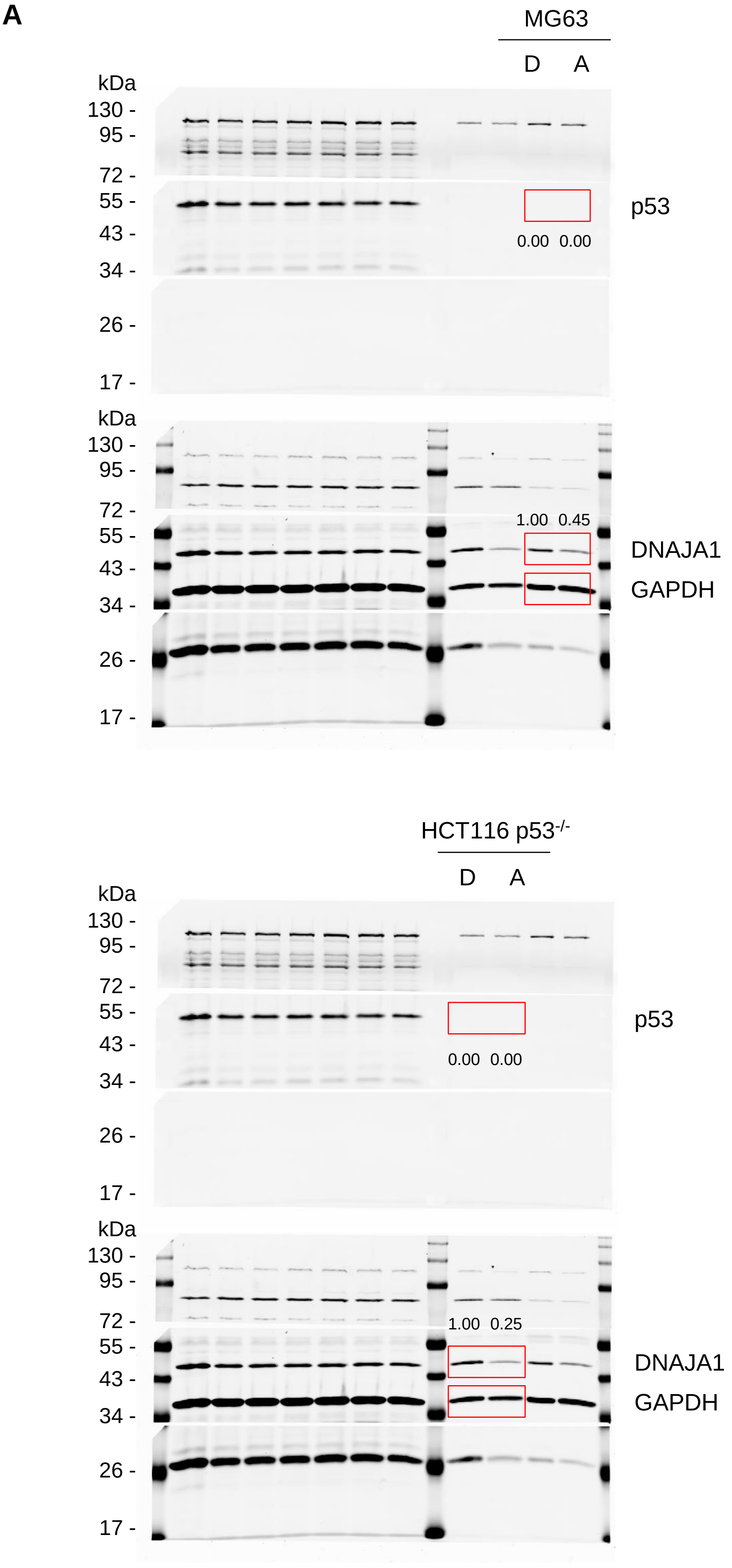

Supplementary Fig S2

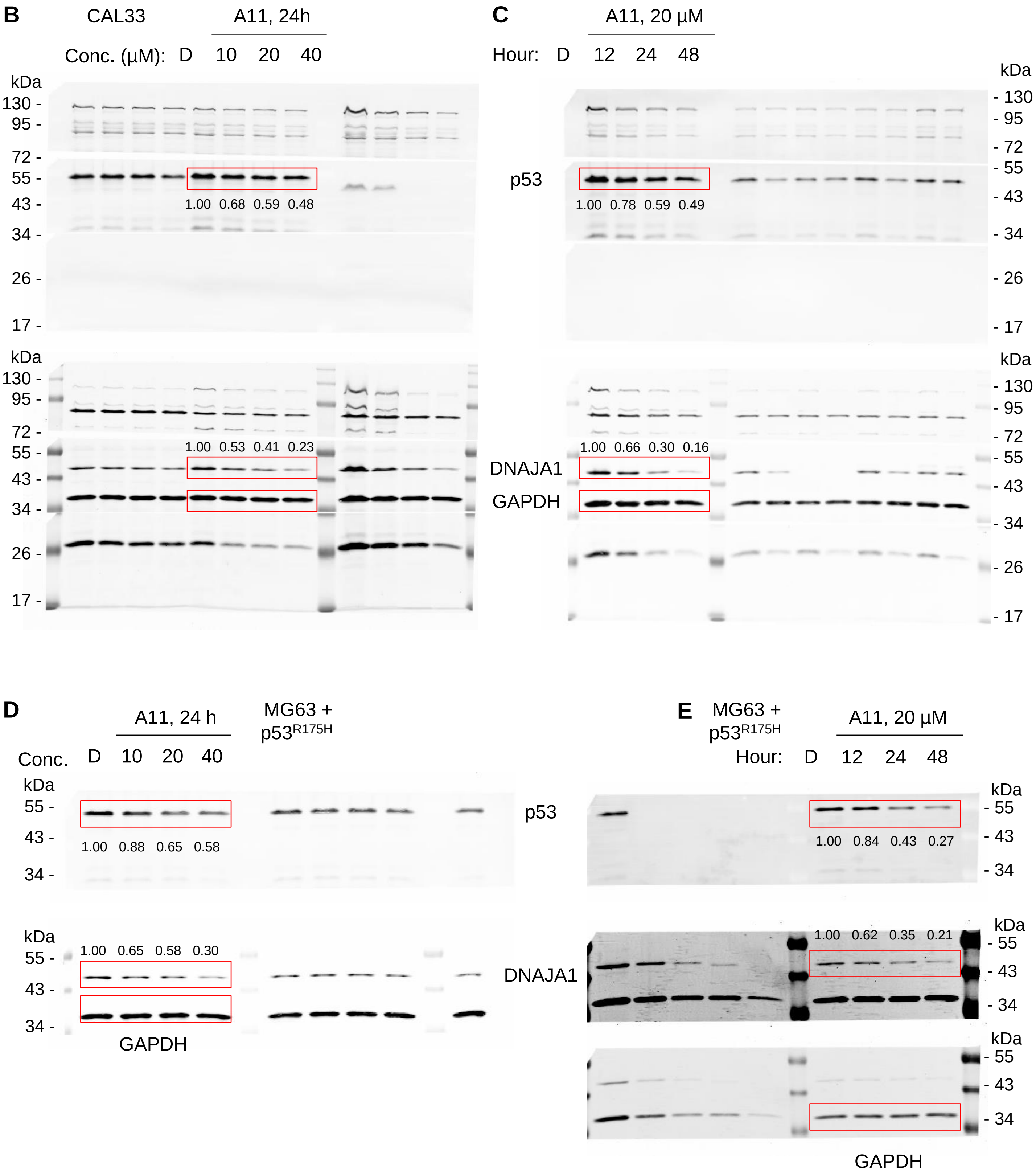

Supplementary Fig S3A

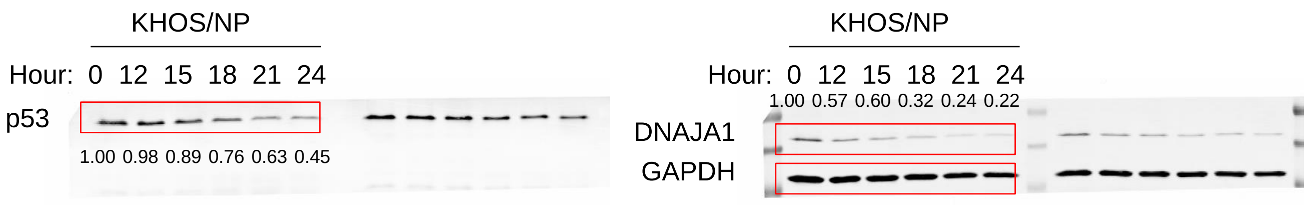

Supplementary Fig S3B

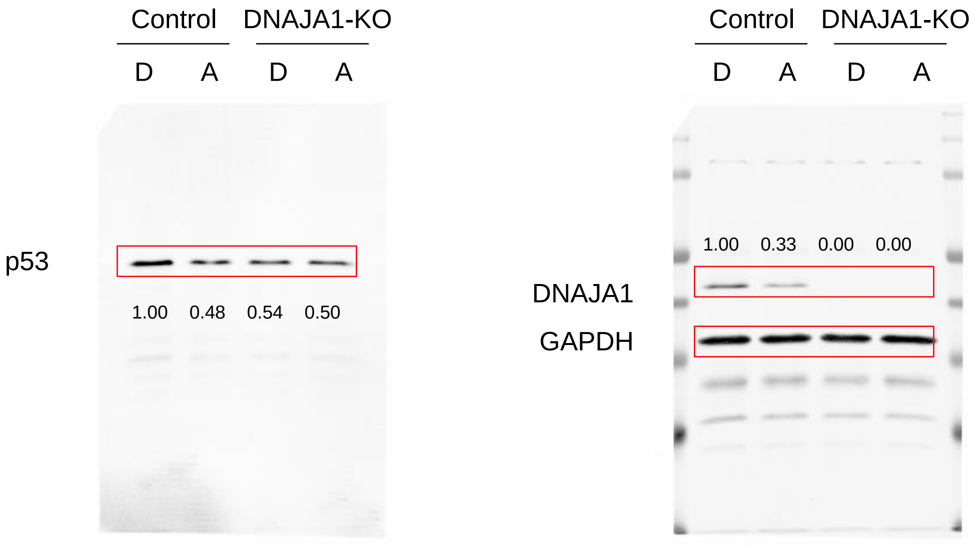

Supplementary Fig S3C

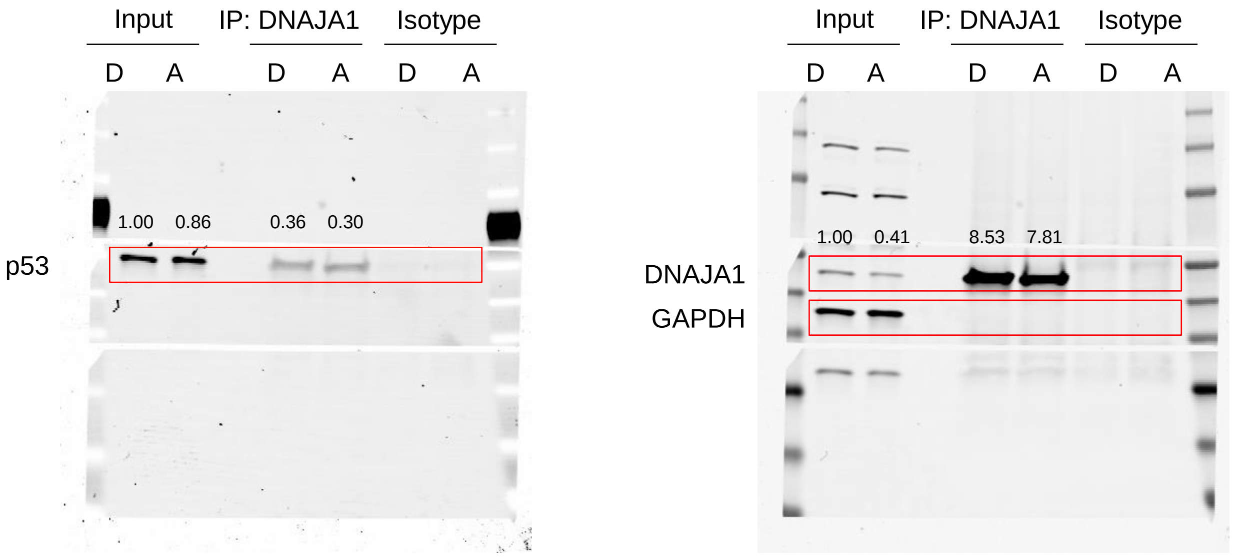

Supplementary Fig S5A

CAL33

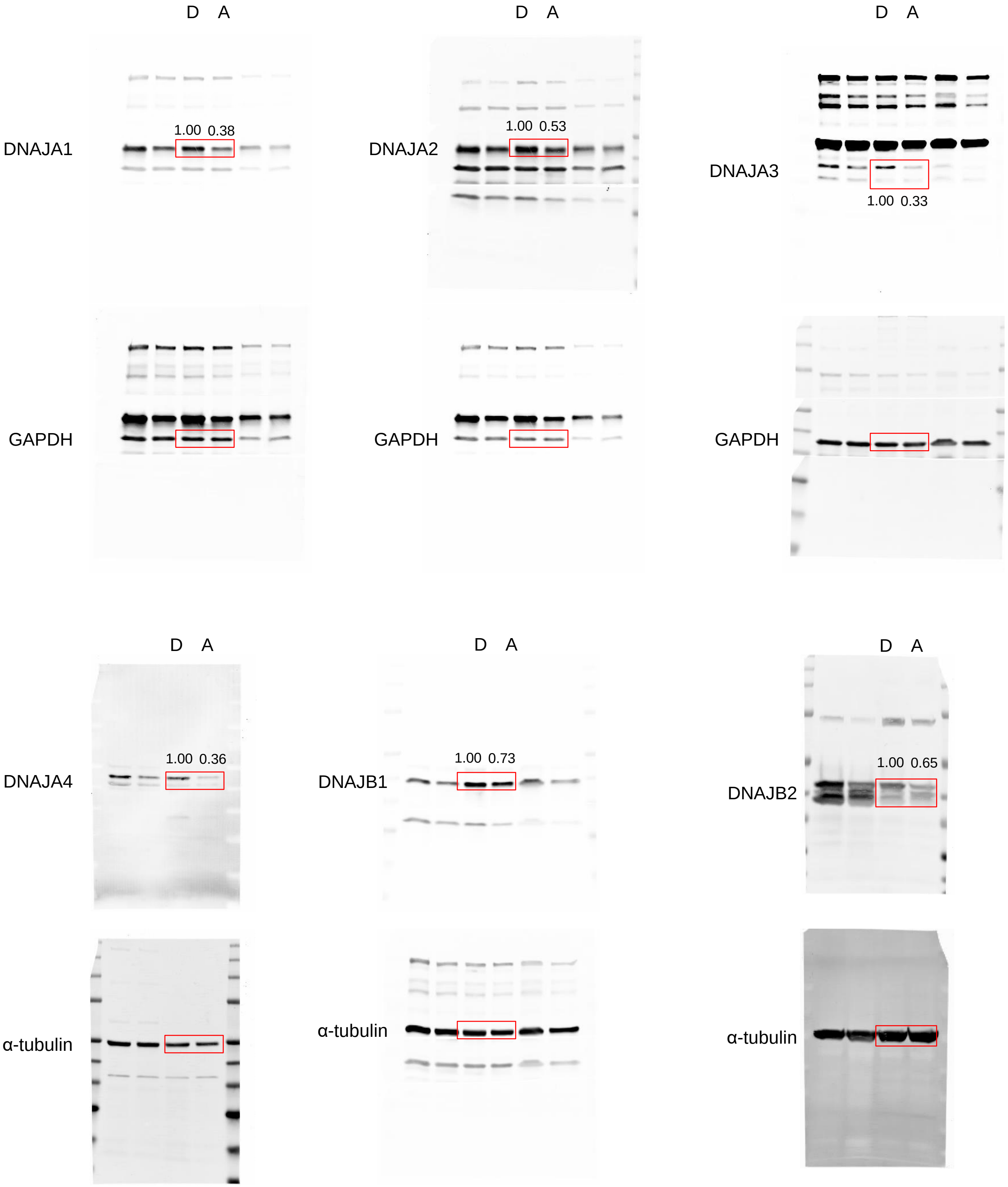

Supplementary Fig S5A

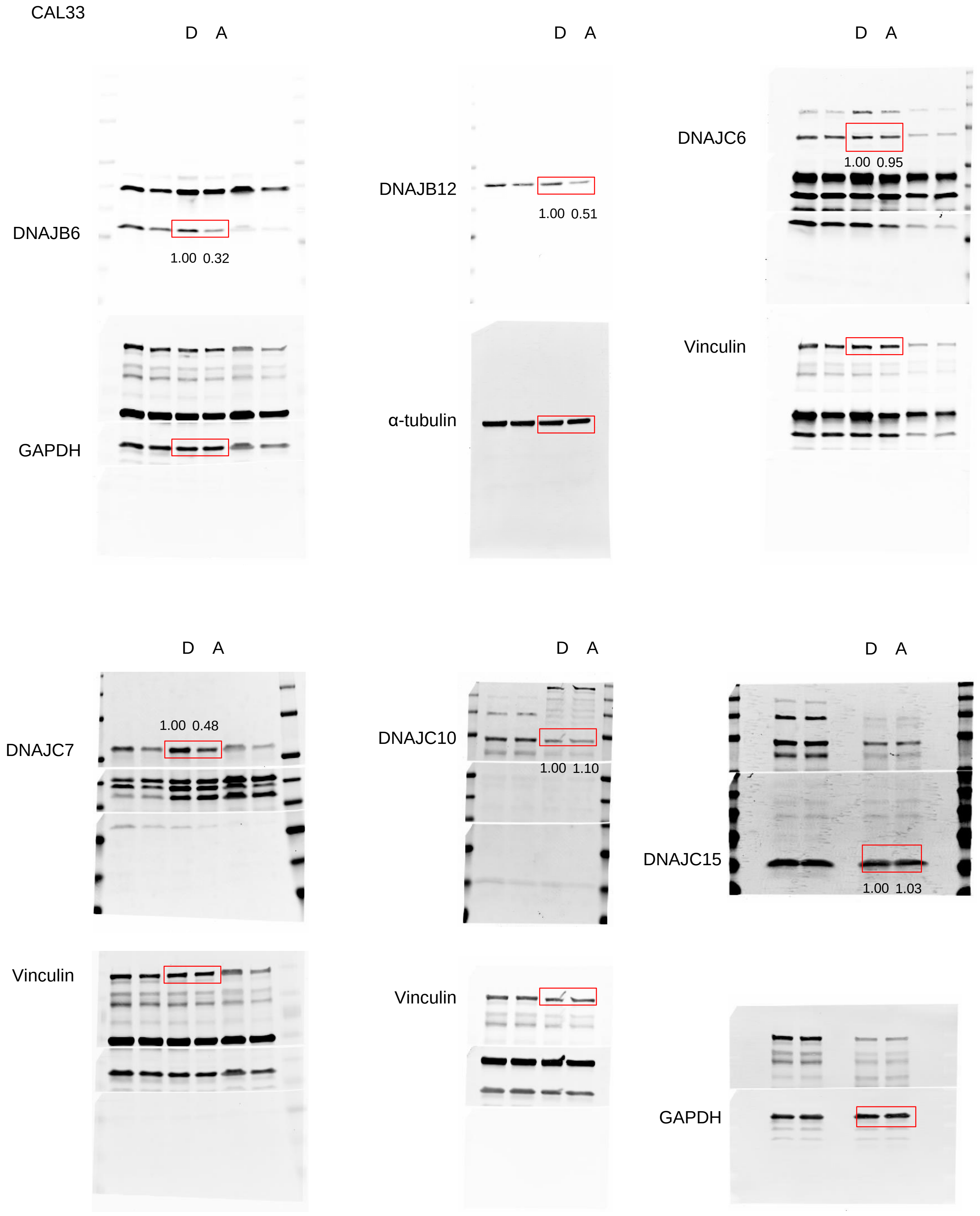

Supplementary Fig S5B

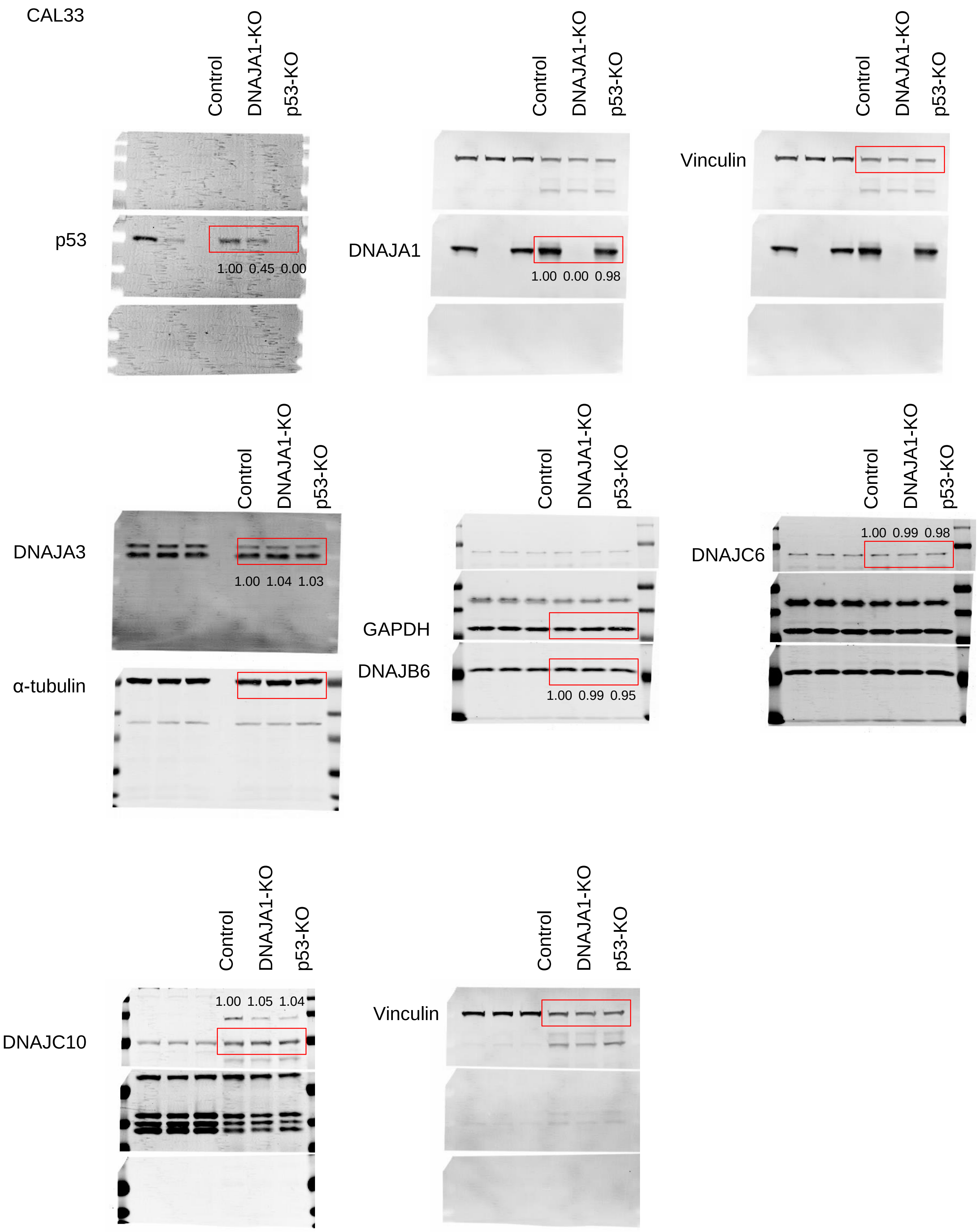

Supplementary Fig S6

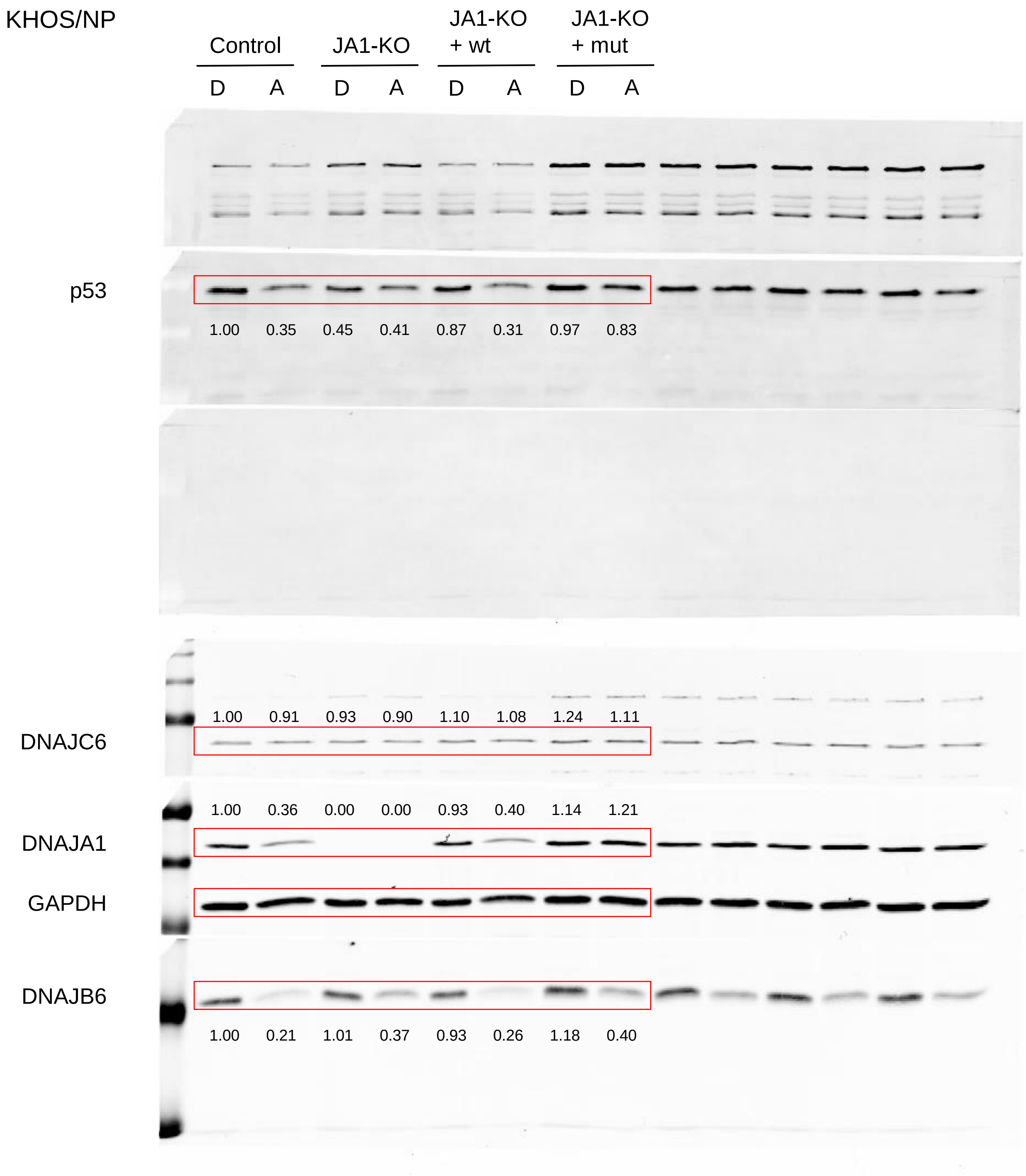

Supplement: Supplementary file 4 — Original Data File [file 41420_2022_1229_MOESM4_ESM.pdf]
