# Supplementary material for: DNAJA1- and conformational mutant p53-dependent inhibition of cancer cell migration by a novel compound identified through a virtual screen
Source: Cell Death Discov. 2022 Oct 31;8:437. doi: 10.1038/s41420-022-01229-5 (PMC9622836; doi:10.1038/s41420-022-01229-5)
Supplement: Supplementary file 5 — Authors list [file 41420_2022_1229_MOESM5_ESM.pdf]

Shigeto Nishikawa s.nskw@kuhp.kyoto-u.ac.jp
Department of Pediatrics, Division of Hematology & Oncology, Children's Mercy Research Institute, Kansas City, MO 64108, USA

Atsushi Kaida koimdth@tmd.ac.jp ORCID ID: 0000-0001-5891-4348
Department of Dental Radiology and Radiation Oncology, Graduate School of Medical and Dental Sciences, Tokyo Medical and Dental University, Tokyo 113-8510, Japan

Alejandro Parrales aparralesbriones@cmh.edu
Department of Pediatrics, Division of Hematology & Oncology, Children's Mercy Research Institute, Kansas City, MO 64108, USA

Atul Ranjan aranjan@cmh.edu ORCID ID: 0000-0003-2920-5707
Department of Pediatrics, Division of Hematology & Oncology, Children's Mercy Research Institute, Kansas City, MO 64108, USA

Mohamed Alalem maalalem@cmh.edu ORCID ID: 0000-0003-2626-295X
Department of Pediatrics, Division of Hematology & Oncology, Children's Mercy Research Institute, Kansas City, MO 64108, USA

Hongyi Ren hren@cmh.edu
Department of Cancer Biology, University of Kansas Medical Center, Kansas City, KS 66010, USA

Frank J. Schoenen schoenen@ku.edu
Higuchi Biosciences Center, University of Kansas, Lawrence, KS 66047, USA

David K. Johnson dkjohnson@ku.edu
Molecular Graphics and Modeling Laboratory, University of Kansas, Lawrence, KS 66045, USA.

Tomoo Iwakuma tiwakuma@cmh.edu ORCID ID: 0000-0001-7249-7958
Department of Pediatrics, Division of Hematology & Oncology, Children's Mercy Research Institute, Kansas City, MO 64108, USA
Department of Cancer Biology, University of Kansas Medical Center, Kansas, KS, USA
